# Enhancing colistin efficacy against *Salmonella* infections with a quinazoline-based dual therapeutic strategy

**DOI:** 10.1038/s41598-024-55793-0

**Published:** 2024-03-01

**Authors:** Carlos A. Lobertti, Fernán O. Gizzi, Christian Magni, Analía Rial, José A. Chabalgoity, Lucía Yim, Víctor S. Blancato, Christopher R. M. Asquith, Eleonora García Véscovi

**Affiliations:** 1 Instituto de Biología Molecular y Celular de Rosario, Consejo Nacional de Investigaciones Científicas y Tecnológicas, Universidad Nacional de Rosario, Predio CCT-CONICET Rosario, S2000 Santa Fe, Rosario Argentina; 2 Departamento de Desarrollo Biotecnológico, Facultad de Medicina, Instituto de Higiene, Universidad de La República, Avda. Alfredo Navarro 3051, 11600 Montevideo, Uruguay; 3 School of Pharmacy, Faculty of Health Sciences, University of Eastern Finland, 70211 Kuopio, Finland

**Keywords:** Drug discovery, Microbiology, Molecular biology

## Abstract

Colistin remains one of the last-resort therapies for combating infections caused by multidrug-resistant (MDR) Enterobacterales, despite its adverse nephro- and neuro-toxic effects. This study elucidates the mechanism of action of a non-antibiotic 4-anilinoquinazoline-based compound that synergistically enhances the effectiveness of colistin against *Salmonella enterica*. The quinazoline sensitizes *Salmonella* by deactivating intrinsic, mutational, and transferable resistance mechanisms that enable *Salmonella* to counteract the antibiotic impact colistin, together with an induced disruption to the electrochemical balance of the bacterial membrane. The attenuation of colistin resistance via the combined treatment approach also proves efficacious against *E. coli*, *Klebsiella*, and *Acinetobacter* strains. The dual therapy reduces the mortality of *Galleria mellonella* larvae undergoing a systemic *Salmonella* infection when compared to individual drug treatments. Overall, our findings unveil the potential of the quinazoline-colistin combined therapy as an innovative strategy against MDR bacteria.

## Introduction

Antibiotic resistance is one of the most important threats to human health worldwide and it is concomitant with the urgency of developing new effective antimicrobial compounds. The acquisition of antibiotic resistance mechanisms by bacteria has been attributed to the selective pressure imposed by environmental, human and animal healthcare, general overuse and misuse of these compounds, and to the scarcity in drug development by the pharmaceutical industry^1,2^.

Bacterial infections are commonly treated with bacteriostatic or bactericidal drugs. Although very effective, alternatives, such as the development of anti-virulence agents that block key pathogenesis mechanisms (pathoblockers), are promising strategies to diminish the emergence of antibiotic resistance mechanisms^3^. In addition, alternative new drugs can act as adjuvants and reduce the effective concentration of commonly used antibiotics and minimize the emergence of resistance.

In this context, the WHO has classified *Salmonella* as a high-priority group for the development of new antibacterial therapies^4,5^. *Salmonella* senses environmental changes and responds to the detection of host cues by triggering a virulence program. PhoP/PhoQ is a signal transduction cascade that belongs to the bacterial family of two-component regulatory systems (TCS) and has an essential role during the infection of *Salmonella* into the mammalian host. This TCS consists of PhoQ, a transmembrane sensor with bifunctional histidine kinase/phosphatase activity, and PhoP, a cytoplasmic response regulator^6^. The system responds to input signals that bacteria encounter during their life cycle, such as availability of Mg^2+^, presence of long chain unsaturated fatty acids or cationic antimicrobial peptides (CAMPs) and acidic pH^7–9^ As adaptive output responses, PhoP/PhoQ controls the expression of genes involved in magnesium homeostasis^10,11^, LPS-modifications^12^, resistance to acidic pH^13^ the internalization and survival within either phagocytic or non-phagocytic cells^14^.

The PhoP/PhoQ system has a crucial role during the life cycle of *Salmonella* and the absence of TCS in mammals, make this TCS an optimal target to develop new antimicrobial therapies^15^. In this regard, we have recently identified two quinazoline-based compounds which negatively regulate PhoP-activated genes by targeting the PhoQ histidine-kinase activity^16^.

Colistin is a member of the polymyxins that are cationic antimicrobial oligopeptides composed of a cyclic heptapeptide linked to a tripeptide acylated at its N-terminus by a fatty acid^17^. These antibiotics disorganize the bacterial envelope in two stages: (1) they interact electrostatically with the negative charges of lipid A, displacing Ca^2+^ and Mg^2+^ that stabilize LPS, and (2) the acylated chain inserts within the outer membrane (OM) with formation of pores, promoting the own entry of the molecule into the periplasm. Then, the antibiotic accesses the inner membrane (IM) by including hydrophilic groups in the fatty acid chains^18^. In addition, Sabnis et al^19^ demonstrated that modified LPS precursors that would be transported from the IM to the OM are also target of colistin, leading to cell lysis^17^. Polymyxin E, known as colistin, is a potent clinical antibiotic whose use nearly ceased in the early 1990s due to nephrotoxicity and neurotoxicity. In recent years, it has returned as a last resort antibiotic, due to its effectiveness against multi-drug resistant Gram-negative pathogens^17^. The veterinary use of colistin allows for the selection of of colistin resistance acquisition in zoonotic bacteria strains, such as non-typhoidal serovars of *Salmonella enterica*, and this mediates the spread of resistance mechanisms along the food chain^20^.

PhoP/PhoQ TCS activates the expression of genes that introduce modifications in LPS. This allows bacteria to counteract the bactericidal effect of polymyxins. In the presence of sub-inhibitory concentrations of colistin, the system promotes the expression of *pmrD*, whose gene product activates the PmrA/PmrB TCS and induces polymyxin resistance genes (*ugd*, *pmrCAB*, *pgbPE*)^12^.

Lipid A is the LPS target of the PhoP/PhoQ-dependent modifications: (1) introduction of 4-amino-4-deoxy-L-arabinose (L-Ara4N) in the phosphate at position 4, controlled by the *pmr*, *arn* or *pbg* operons^12^ and (2) introduction of phosphoethanolamine (PEtN) in the position 1-phosphate, controlled by *pmrC*^21,22^ Ugd, an UDP-glucose dehydrogenase under PhoP, PmrA and Rcs-dependent regulation, plays a crucial role in L-Ara4N pathway since it converts UDP-glucose to UDP-glucuronic acid, the substrate for the *arn* pathway^23,24^.

In this study, we demonstrate how the quinazoline CA439 attenuates the expression of genes that define colistin resistance and thus, it increases colistin efficacy. The quinazoline-mediated enhancement of the polymyxin antibiotic capacity does not only rely on the PhoP/PhoQ-dependent resistance mechanisms inactivation but also on the concomitant alteration of electrochemical properties of the bacterial membrane. The dual treatment attenuates *Salmonella* virulence in an in vivo model of infection. In sum, our results position the combined colistin-quinazoline use as a promising option to develop a therapeutic strategy to fight against *Salmonella* infections.

## Results

### CA439 potentiates the bactericidal effect of colistin

**Figure 1:**
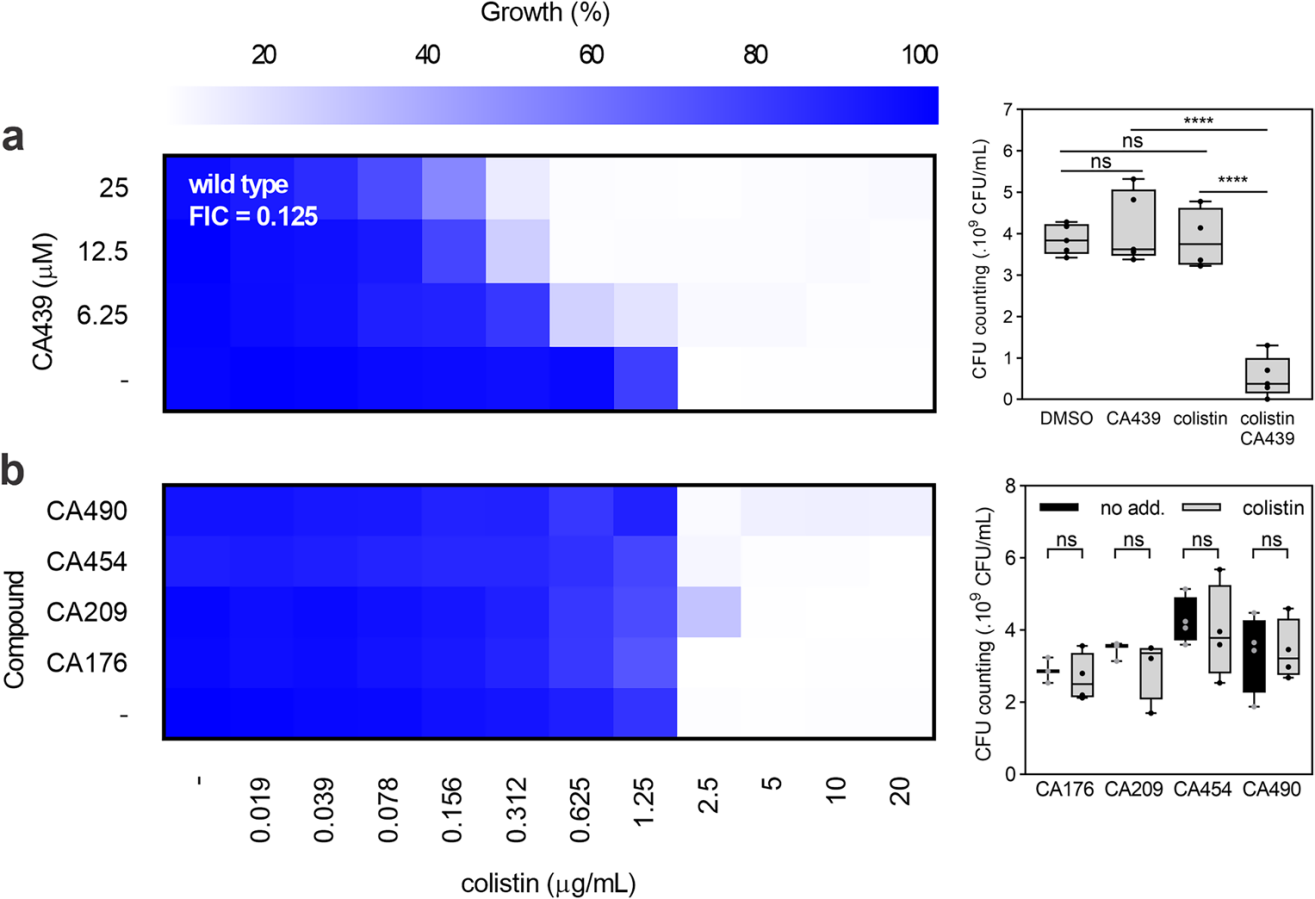
Susceptibility to colistin can be increased by combination with CA439. Checkerboard assay showing the combination of colistin with (**a**) increasing doses of CA439 or (**b**) 25 μM of CA-analogues that do not inhibit the PhoP/PhoQ system. Dark blue regions represent higher cell density. Data represent the mean OD (600 nm) of at least three biological replicates. Right panel shows the CFU counting obtained in each growth condition: (**a**) DMSO (LB-medium with the addition of DMSO), CA439 (25 μM CA439 alone), colistin (0.312 μg mL^−1^ colistin alone) and CA439 + colistin (25 μM CA439 + 0.312 μg mL^−1^ colistin) and (**b**) 25 μM of each compound with or without 1.25 μg mL^−1^ colistin. Fractional inhibitory concentration (FIC) was obtained by dividing the colistin MIC in the presence of CA439 (0.312 μg mL^−1^) by the colistin MIC in the absence of CA439 (2.5 μg mL^−1^). Statistical analysis was performed (**a**) using one-way ANOVA with Tukey’s correction multiple comparison test (****P < 0.0001; ns, no significant differences) and (**b**) unpaired *t* test for each compound assayed (ns, no significant difference).

*Salmonella typhimurium* ATCC 14028 (STM14028) was used as a study model throughout this work. 4-anilinoquinazoline-based compounds down-regulate the activity of the *S. typhimurium* PhoP/PhoQ system by acting as competitive inhibitors of ATP in the PhoQ autophosphorylation reaction^16^. We selected CA439 (GI262866A) as representative of these quinazolines (synthesized as previously described^16^) to evaluate its ability to potentiate colistin activity. All assays were performed in LB, a low Mg^2+^ medium that ensures the PhoP/PhoQ system with an inducing environmental condition^7–10^. CA439 effect was evaluated up to 25 μM, a sub-inhibitory concentration for *Salmonella* growth capacity that showed a repressive effect over PhoP/PhoQ activity^16^. The checkerboard assay was employed in which the fractional inhibitory concentration (FIC) was calculated^25^. The dose-dependent enhancing effect of colistin by CA439 reduced the MIC of colistin from 2.5 μg mL^−1^ to 0.312 µg mL^−1^, and resulted in a FIC of 0.125 (Fig. 1a, left). This was corroborated by counting colony-forming units (Fig. 1a, right). None of the CA439-analogues (CA176, CA209, CA454 and CA490, previously synthesized and characterized^16^) that exerted no repressive effect over PhoP/PhoQ activity showed a colistin-enhancing effect (Fig. 1b), reinforcing that the effect of CA439 selectively targets PhoQ histidine kinase.

CA439 is able to access the bacterial cytoplasm as it interacts with the catalytic domain of PhoQ^16^. Despite this, we hypothesized if a permeabilizing effect of sublethal concentrations of colistin could allow to build-up an intracellular concentration of quinazoline that was lethal to *Salmonella*. No potentiating effect was observed when CA439 and either EDTA or SDS (OM-permeabilizing agents that do not promote LPS modifications) were combined (Supplementary Fig. S1), suggesting that CA439-colistin potentiation does not rely on the colistin-mediated entry of the quinazoline by an OM permeabilizing action.

### CA439-colistin enhancing effect is partially PhoP/PhoQ-dependent

**Figure 2:**
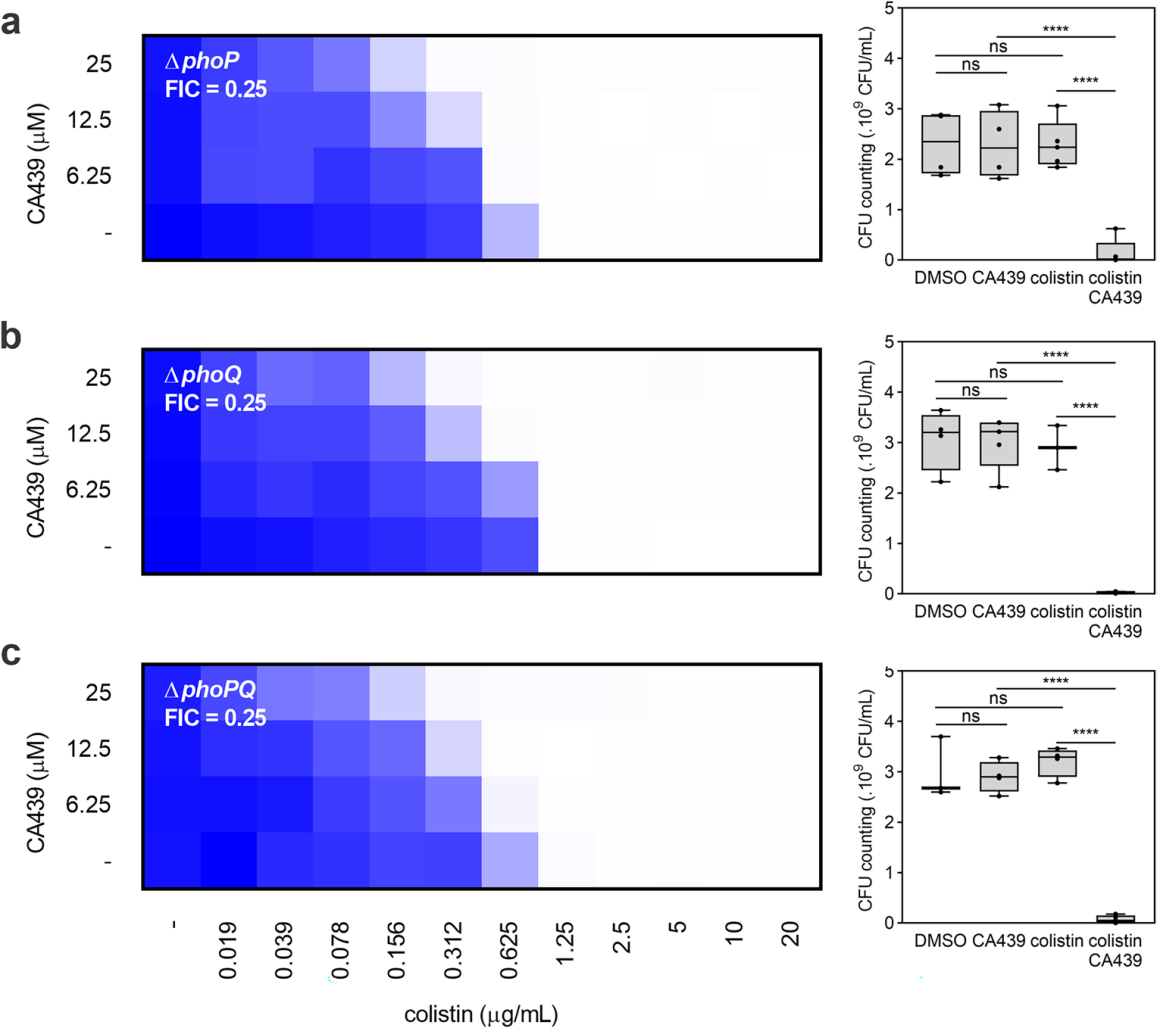
CA439 enhancement of the bactericidal effect of colistin is partially PhoP/PhoQ-dependent. Checkerboard assay showing CA439-colistin combination in (**a**) *phoP* mutant, (**b**) *phoQ* mutant and (**c**) *phoPQ* mutant. Dark blue regions represent higher cell density. Data represent the mean OD (600 nm) of at least three biological replicates. Right panel shows the CFU counting obtained in each growth condition: DMSO (LB-medium with the addition of DMSO), CA439 (25 μM CA439 alone), colistin (0.312 μg mL^−1^ colistin alone) and CA439 + colistin (25 μM CA439 + 0.312 μg mL^−1^ colistin). Fractional inhibitory concentration (FIC) was obtained by dividing the colistin MIC in the presence of CA439 (0.312 μg mL^−1^) by the colistin MIC in the absence of CA439 (1.25 μg mL^−1^). Statistical analysis was performed using one-way ANOVA with Tukey’s correction multiple comparison test (**** *P* < 0.0001; ns, no significant differences).

To assess whether the observed phenotype depends exclusively on the inhibition of the PhoP/PhoQ system by CA439, single mutants in the response regulator (Δ*phoP*) or in the histidine kinase (Δ*phoQ*), and a Δ*phoPQ* mutant strain were tested (Fig. 2). The colistin-CA439 enhancing phenotype was reduced by twofold in these strains (a FIC of 0.25 for mutant strains versus a FIC of 0.125 for the wild type strain), indicating that CA439 exerts an additional action alongside with the effect on PhoP/PhoQ. Both mutant strains were tested in the presence of CA439 inactive analogues, and no potentiation effect was observed (Supplementary Fig. S2), indicating that the CA439 enhancing effect is specific to this compound.

### CA439 effect on the PmrA/PmrB activity depends on PhoP/PhoQ integrity

**Figure 3:**
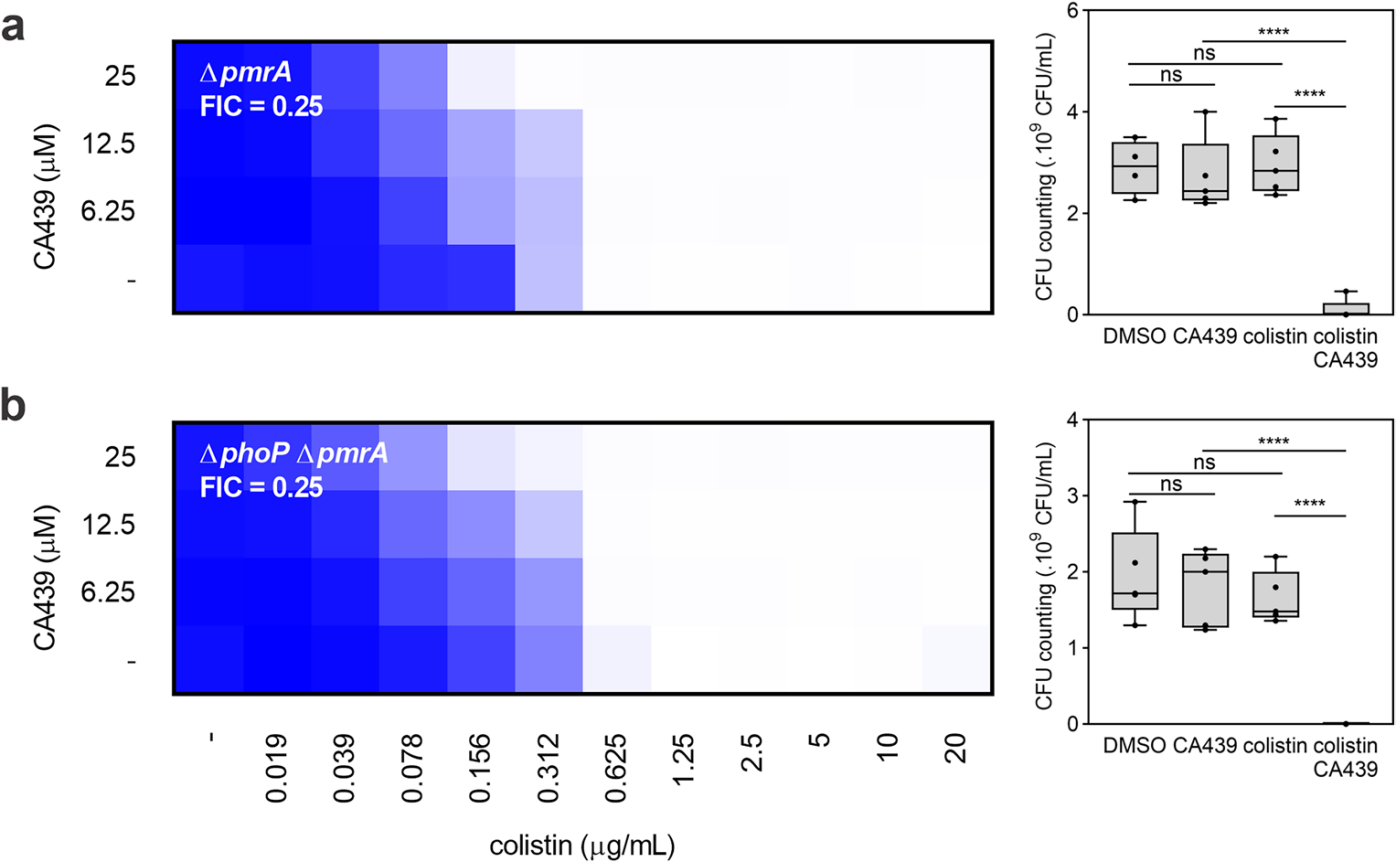
CA439-colistin potentiation indirectly affects PmrA/PmrB system activity. Checkerboard assay showing CA439-colistin combination in (**a**) *pmrA* mutant or (**b**) *phoP pmrA* double mutant. Dark blue regions represent higher cell density. Data represent the mean OD (600 nm) of at least three biological replicates. Right panel shows the CFU counting obtained in each growth condition: DMSO (LB-medium with the addition of DMSO), CA439 (25 μM CA439 alone), colistin (0.156 μg mL^−1^ colistin alone) and CA439 + colistin (25 μM CA439 + 0.156 μg mL^−1^ colistin). Fractional inhibitory concentration (FIC) was obtained by dividing the colistin MIC in the presence of CA439 (0.156 μg mL^−1^) by the colistin MIC in the absence of CA439 (0.625 μg mL^−1^). Statistical analysis was performed using one-way ANOVA with Tukey’s correction multiple comparison test (**** *P* < 0.0001; ns, no significant differences).

PhoP/PhoQ and PmrA/PmrB TCS are functionally interconnected^12^ and they together activate the expression of genes that confer resistance to polymyxins. To assess whether PmrA/PmrB is directly targeted by CA439 and independently of PhoP/PhoQ, we analyzed Δ*pmrA* and double Δ*phoP* Δ*pmrA* mutant strains (Fig. 3). Both mutants behaved similarly to the Δ*phoPQ* strain (FIC of 0.25), consistent with the fact that PmrA/PmrB activity is subordinated to the PhoP/PhoQ TCS^12^.

**Figure 4:**
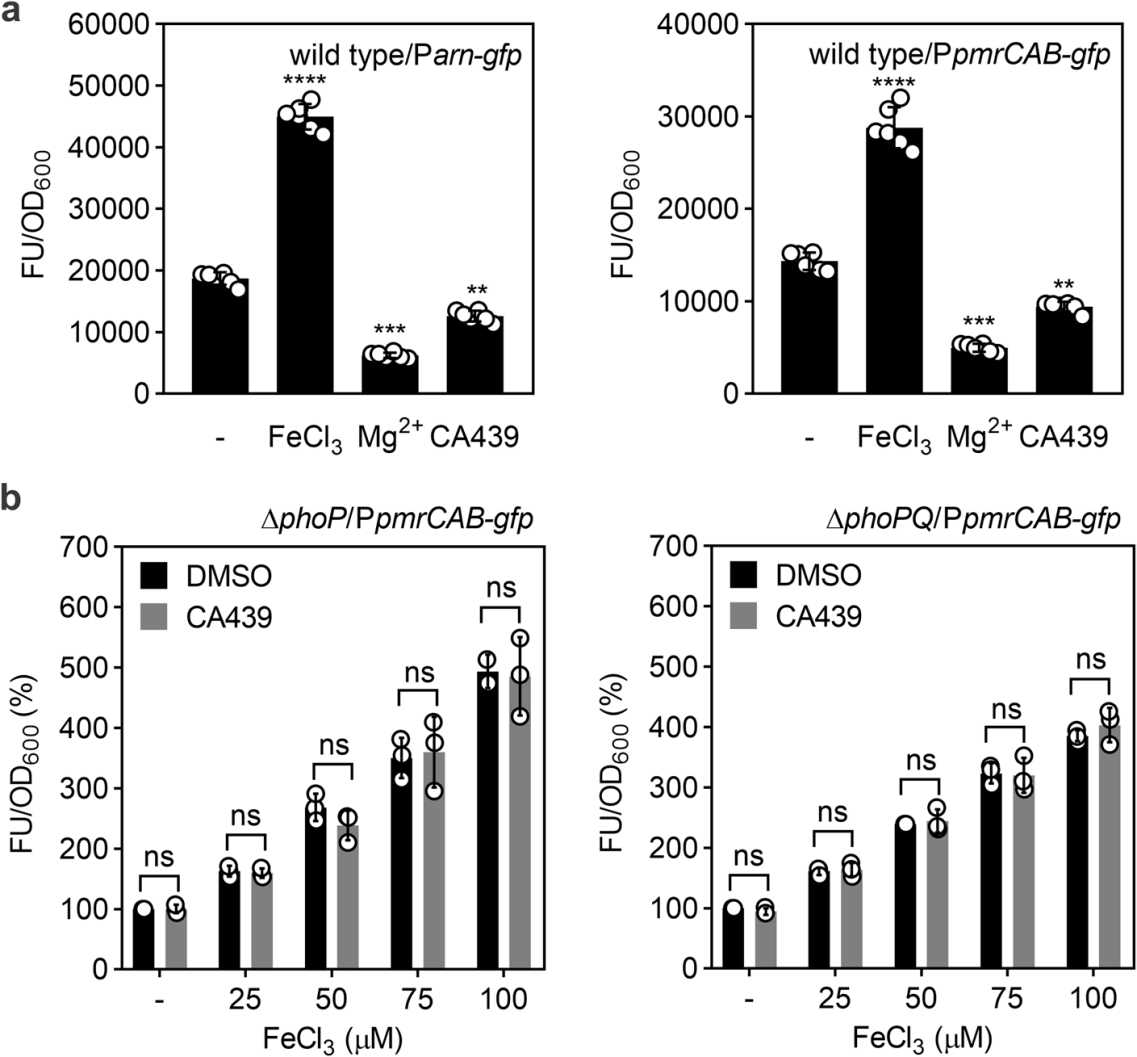
CA439 inhibitory effect on PmrA/PmrB activity depends on PhoP/PhoQ. (**a**) Transcriptional activity from *arn::gfp* (left) or *pmrCAB::gfp* (right) fusions in the wild type strain grown in the presence of 100 μM FeCl_3_, 5 mM MgCl_2_ or 25 μM CA439. (**b**) Transcriptional activity from *pmrCAB::gfp* fusion in a *phoP* (left) or *phoPQ* (right) mutant strain background grown in the presence of increasing concentrations of FeCl_3_ (25, 50, 75, 100 μM) with or without the addition of 25 μM CA439. Bacteria were grown at 37 °C for 16 h in the condition indicated in each assay. Transcriptional activity was calculated from the ratio between the fluorescence values (FU) and OD_600_ values (FU/OD_600_). The results represent the average of at least three independent experiments and error bars correspond to standard deviation (SD). Statistical analysis was performed using a, one-way ANOVA with Tukey’s correction multiple comparison test (****P < 0.0001, ***P < 0.001, **P < 0.01) or (**b**) unpaired *t* test (ns, no significant differences).

To confirm that CA439 does not modulate PmrA/PmrB directly, we evaluated whether the compound could modulate the expression of L-Ara4N and PEtN modifications via PmrA/PmrB and independently of PhoP/PhoQ. *arn* or *pmrCAB* plasmidic transcriptional reporters were constructed by cloning the promoter regions of either operons upstream *gfp* that encodes the green fluorescent protein (GFP). The reporters were tested into the wild type, the Δ*phoP* or the Δ*phoPQ* mutant backgrounds, in the presence or absence of FeCl_3_ (activator of the PmrA/PmrB system)^26^, Mg^2+^ (repressor of the PhoP/PhoQ system)^7^ or CA439. Quinazoline CA439 inhibited the expression of the *arn* and the *pmrCAB* operons only in the presence of an intact *phoPQ* operon (Fig. 4a). No modulation of the fluorescence levels was detected in the Δ*phoP* or Δ*phoPQ* backgrounds, irrespective of the level of PmrA/PmrB induction achieved by addition of increasing concentrations of FeCl_3_ in the growth medium (Fig. 4b). These results rule out the direct effect of CA439 onto the PmrA/PmrB TCS system activity.

### CA439-colistin potentiation phenotype partially depends on L-Ara4N and PEtN pathways

**Figure 5:**
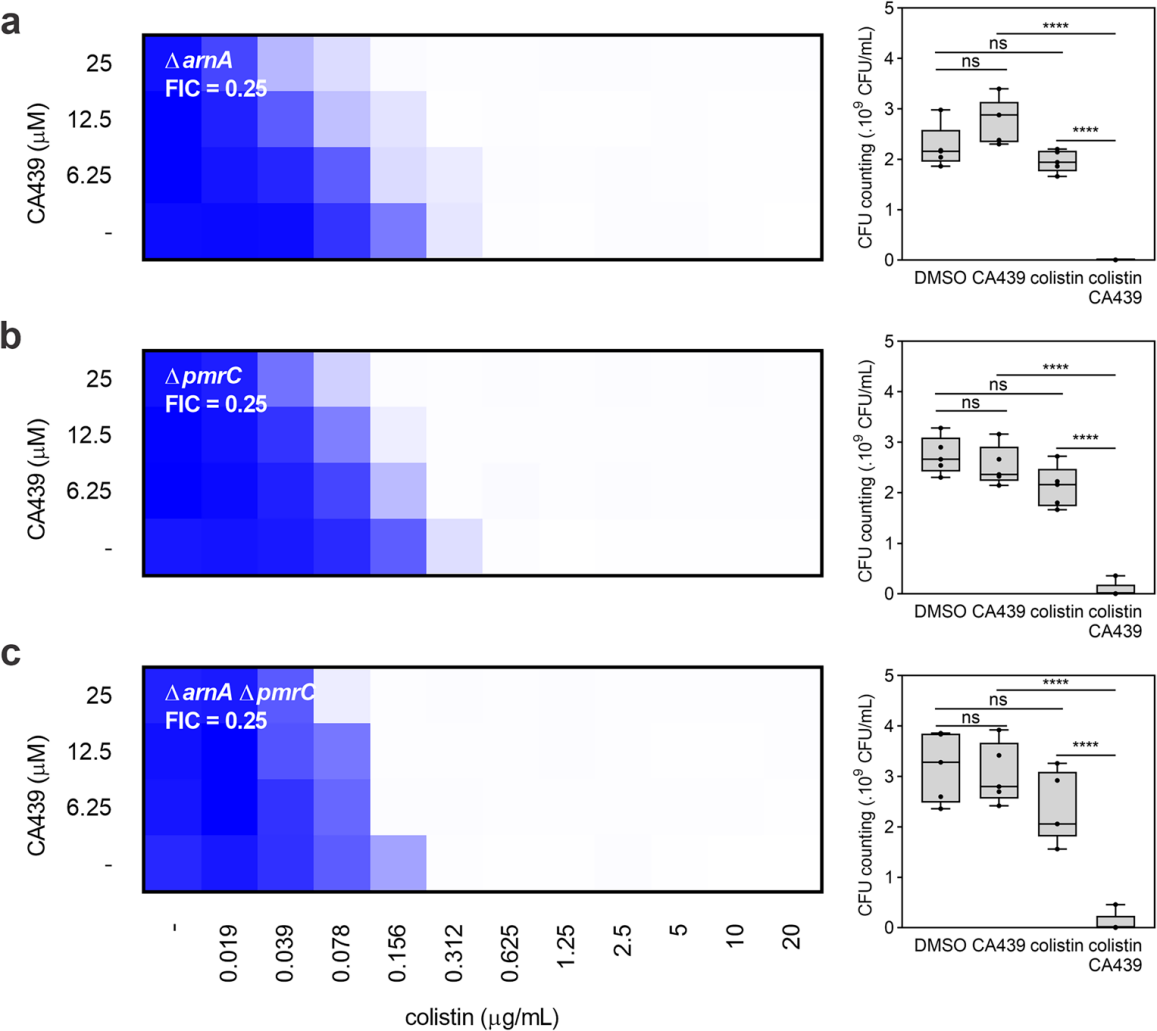
L-Ara4N and PEtN pathways disruption does not completely reverse CA439-colistin potentiation. Checkerboard assay showing CA439-colistin combination in (**a**) *arnA* mutant, (**b**) *pmrC* mutant or **c,** *arnA pmrC* double mutant strains. Dark blue regions represent higher cell density. Data represent the mean OD (600 nm) of at least three biological replicates. Right panel shows the CFU counting obtained in each growth condition: DMSO (LB-medium with the addition of DMSO), CA439 (25 μM CA439 alone), colistin (0.078 μg mL^−1^ colistin alone) and CA439 + colistin (25 μM CA439 + 0.078 μg mL^−1^ colistin). Fractional inhibitory concentration (FIC) was calculated by dividing the colistin MIC in the presence of CA439 (0.078 μg mL^−1^) by the colistin MIC in the absence of CA439 (0.312 μg mL^−1^). Statistical analysis was performed using one-way ANOVA with Tukey’s correction multiple comparison test (****P < 0.0001; ns, no significant differences).

To explore whether CA439 might impact a PhoP/PhoQ and PmrA/PmrB independent pathway that modulates LPS modifications, we constructed mutant strains in *arnA*^24^ and *pmrC*, which disrupt L-Ara4N and PEtN lipid A modifications. Quinazoline CA439 combined with colistin was evaluated in a Δ*arnA*, Δ*pmrC* and Δ*arnA* Δ*pmrC* mutant backgrounds (Fig. 5). The inactivation of these two main LPS modification pathways was not sufficient to abrogate the enhancer phenotype, confirming that CA439 has an additional, PhoP/PhoQ-PmrA/PmrB-independent, effect.

### The CA439-colistin potentiation is partially Rcs-dependent

Membrane stress induced by CAMPs is also sensed by the Rcs TCS^23,27^. Rcs controls several cellular processes, among which is the synthesis of colanic acid (CA) that results in the production of capsular polysaccharide and the activation of *ugd* that is required for both CA biosynthesis and L-Ara4N modification of LPS^23^. To analyze this, we evaluated the effect of inactivating *wcaL*, *wzxc*, *wzb* or *wcaJ*, Rcs-dependent genes involved in CA synthesis^28^. Their inactivation did not alter the MIC value of colistin when compared with the wild type strain (Supplementary Figs. S3 and S4), indicating that the products of these genes have no role in the resistance mechanism.

**Figure 6:**
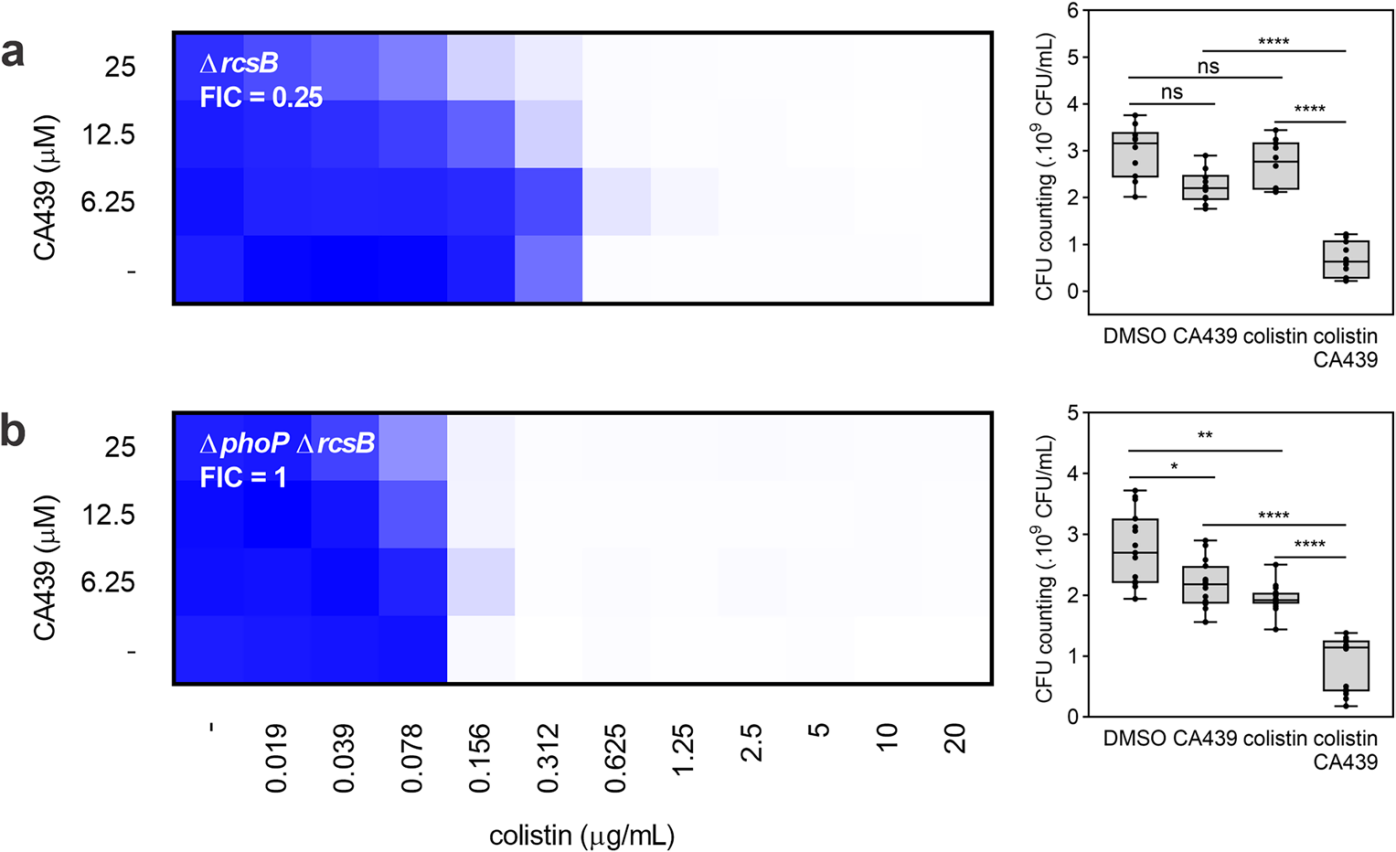
CA439-colistin potentiation is partially Rcs-dependent. Checkerboard assay showing CA439-colistin combination in (**a**) *rcsB* mutant or (**b**) *phoP rcsB* double mutant. Dark blue regions represent higher cell density. Data represent the mean OD (600 nm) of at least three biological replicates. Right panel in shows the CFU counting obtained in each growth condition: DMSO (LB-medium with the addition of DMSO), CA439 (25 μM CA439 alone), colistin (0.156 μg mL^−1^ for Δ*rcsB* and 0.078 μg mL^−1^ for Δ*phoP* Δ*rcsB*) and CA439 + colistin (25 μM CA439 + 0.156 μg mL^−1^ colistin for Δ*rcsB* and 0.078 μg mL^−1^ colistin for Δ*phoP* Δ*rcsB*). Fractional inhibitory concentration (FIC) was obtained by dividing the colistin MIC in the presence of CA439 (0.156 μg mL^-1^ for both strains) by the colistin MIC in the absence of CA439 (0.625 μg mL^−1^ for Δ*rcsB* and 0.156 μg mL^−1^ for Δ*phoP* Δ*rcsB*). Statistical analysis was performed using one-way ANOVA with Tukey’s correction multiple comparison test (****P < 0.0001; **P < 0.001; *P < 0.01; ns, no significant differences).

We then analyzed the behavior of single Δ*rcsB* and a double Δ*phoP* Δ*rcsB* mutant strains in the presence of the CA439-colistin combination (Fig. 6). Δ*rcsB* partially reversed the phenotype (FIC of 0.25 compared to FIC of 0.125 for the wild type strain) (Fig. 6a) while the double mutant strain strongly attenuated the phenotype (Fig. 6b), indicating that there is at least one Rcs regulon member involved in CA439-colistin potentiation effect.

The transcriptional expression of the UDP-glucose dehydrogenase gene (*ugd*) required for both CA biosynthesis and L-Ara4N modification of LPS, depends on PhoP, PmrA and RcsB^23^. Therefore, a single Δ*ugd* and a double Δ*ugd* Δ*pmrC* mutant strains in which CA biosynthesis, L-Ara4N and PEtN modifications are affected were tested (Supplementary Fig. S5). The Δ*ugd* strain showed an eightfold decrease in MIC values and a FIC of 0.5 versus FIC of 0.125 for the wild type strain (Supplementary Fig. S5a). Δ*ugd* Δ*pmrC* and Δ*phoP* Δ*rcsB* strains behaved similarly (Supplementary Fig. S5b and Fig. 6b), indicating that CA439 inhibitory action impacts *ugd* and *pmrC* expression, increasing colistin sensitivity. However, a residual potentiation effect observed in Δ*phoP* Δ*rcsB* and Δ*ugd* Δ*pmrC* strains indicates that an additional mechanism still contributes to the CA439-dependent increased sensitivity to colistin.

### CA439 selectively potentiates polymyxins

Next, we analyzed if the quinazoline could affect other bacterial targets different from colistin. We assayed CA439 combined with conventional antibiotics that act at peptidoglycan level (ampicillin, imipenem, meropenem), at protein translation level (chloramphenicol, kanamycin, azithromycin, tetracycline) or at gene transcription level (rifampicin). There was no alteration in the efficacy of antibiotics with mechanisms that do not involve LPS or the inner bacterial membrane (Supplementary Fig. S6), indicating that the potentiating effect of CA439 is selective for colistin (or for colistin-type compounds).

### CA439 alters membrane polarization and permeabilizes the outer membrane

Therefore, we explored whether CA439 could exert an additional PhoP/PhoQ-PmrA/PmrB and Rcs-independent effect on the bacterial membrane.

**Figure 7:**
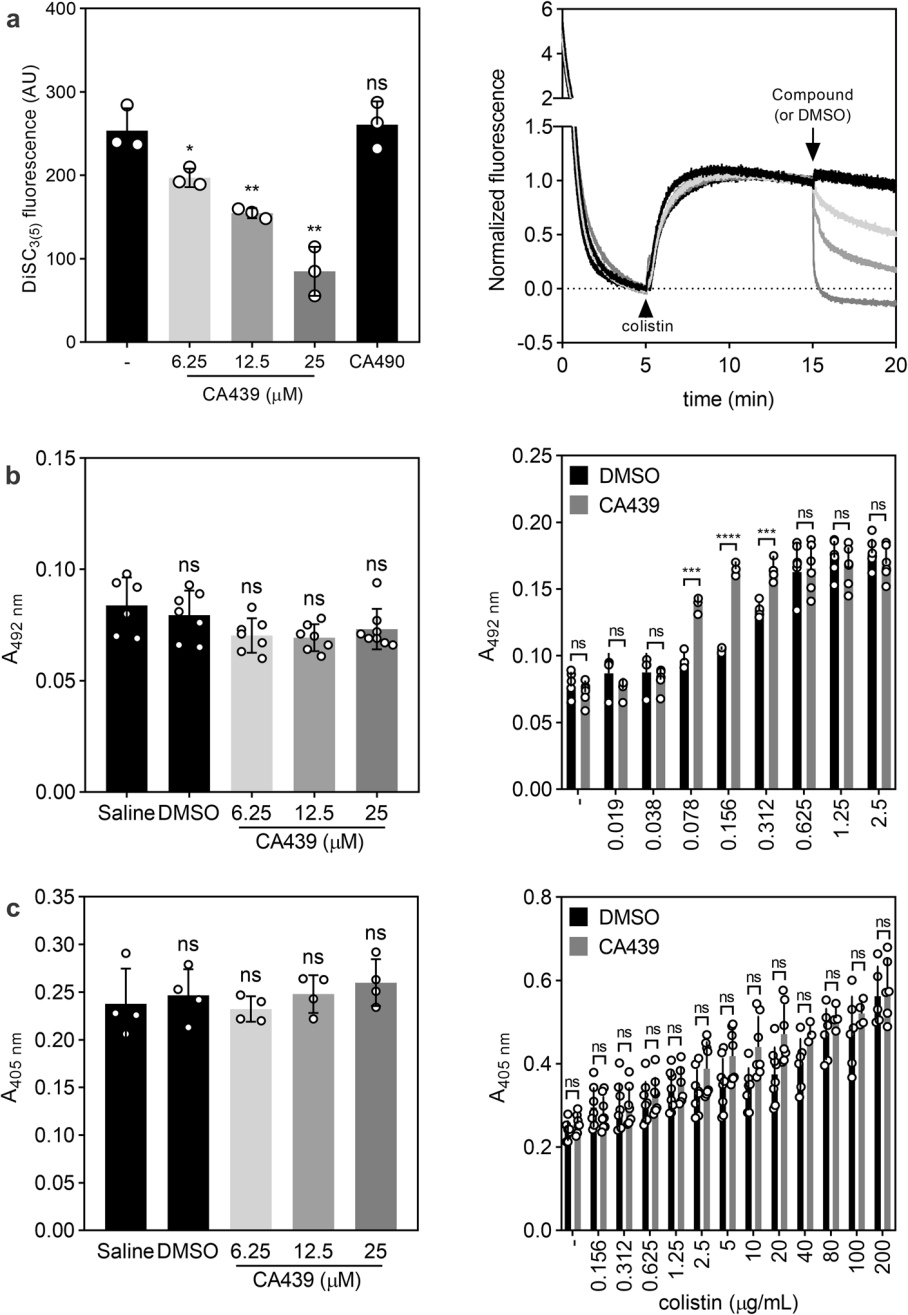
CA439 alters IM potential and promotes colistin-mediated lysis. (**a**) Fluorescence intensity was monitored from a wild type strain culture incubated with 1 μM DiSC_3(5)_ for 5 min and then with 5 μg mL^−1^ colistin for another 10 min followed by the indicated concentration of CA439, 25 μM CA490 or the equivalent volume of DMSO used for the maximum concentration of compound tested. Statistical analysis was performed using one-way ANOVA with Tukey’s correction multiple comparison test against no addition condition (-) (** *P* < 0.001; * *P* < 0.01; ns, no significant differences). (**b**) OM disruption and **c**, IM disruption were measured using β-lactamase assay and β-galactosidase assay, respectively, as described in Materials and Methods. Cells expressing (**b**) β-lactamase or (**c**) β-galactosidase were incubated with PBS (Saline), DMSO or increasing concentrations of CA439 (left panel) or colistin (**b**) from 0 to 2.5 μg mL^−1^ or (**c**) from 0 to 200 μg mL^−1^) in the presence of 25 μM CA439 (grey bars) or DMSO (dark bars) (right panel). (**b**) Nitrocefin (30 μM) or (**c**) ONPG (2 mM) were added as substrate and absorbance at 492 nm and 405 nm, respectively, were monitored. Statistical analysis was performed using unpaired *t* test (**** *P* < 0.0001; *** *P* < 0.001; ns, no significant differences). The results are the average of at least three independent experiments.

The fluorescent probe DiSC_3(5)_ is used to detect differences in membrane potential^29^. The probe is a positively charged and hydrophobic molecule that associates with polarized membranes. The IM of Gram-negative bacteria has a negative electrochemical potential, with accumulation of positive charges in the periplasmic space and negative charges in the intracellular space. On polarized bilayers, DiSC_3(5)_ fluorescence is self-quenched as the molecule associates with the membrane. Alterations in membrane potential will make the probe increase its fluorescence as it migrates from the membrane^29^. Therefore, wild type bacteria were preincubated with the probe until stable basal fluorescence intensity was reached (5 min). After this time, colistin was added first (5 min) and increasing concentrations of either CA439, negative control CA490 or DMSO were subsequently added (Fig. 7a). CA439 promoted a decrease in fluorescence intensity to basal levels, indicating entry of the probe. This shows an effect at the IM level. Consistent with the CA439-specific phenotypes obtained so far, CA490 caused no alteration.

Next, we compared the permeabilization action of colistin and/or CA439 on the bacterial IM versus the OM. We tested the ability of the compounds to allow the entry of two substrates that require a previous permeabilization of the OM or the IM to get in contact with a hydrolytic enzyme: nitrocefin, substrate of periplasmic beta-lactamase, or *o*-nitrophenyl-*β*-galactoside (ONPG), substrate of cytoplasmic beta-galactosidase. While colistin is able to alter both IM and OM integrity^30^, CA439 alone did not disrupt IM integrity and it did not alter OM permeability unless it was combined with colistin, enhancing its effect (Fig. 7b,c).

Together, the results indicate that CA439 promotes the entry of colistin through the OM and exerts an effect on the IM by altering proton motive force homeostasis.

### CA439 shows effectiveness against strains with high resistance to colistin

**Figure 8:**
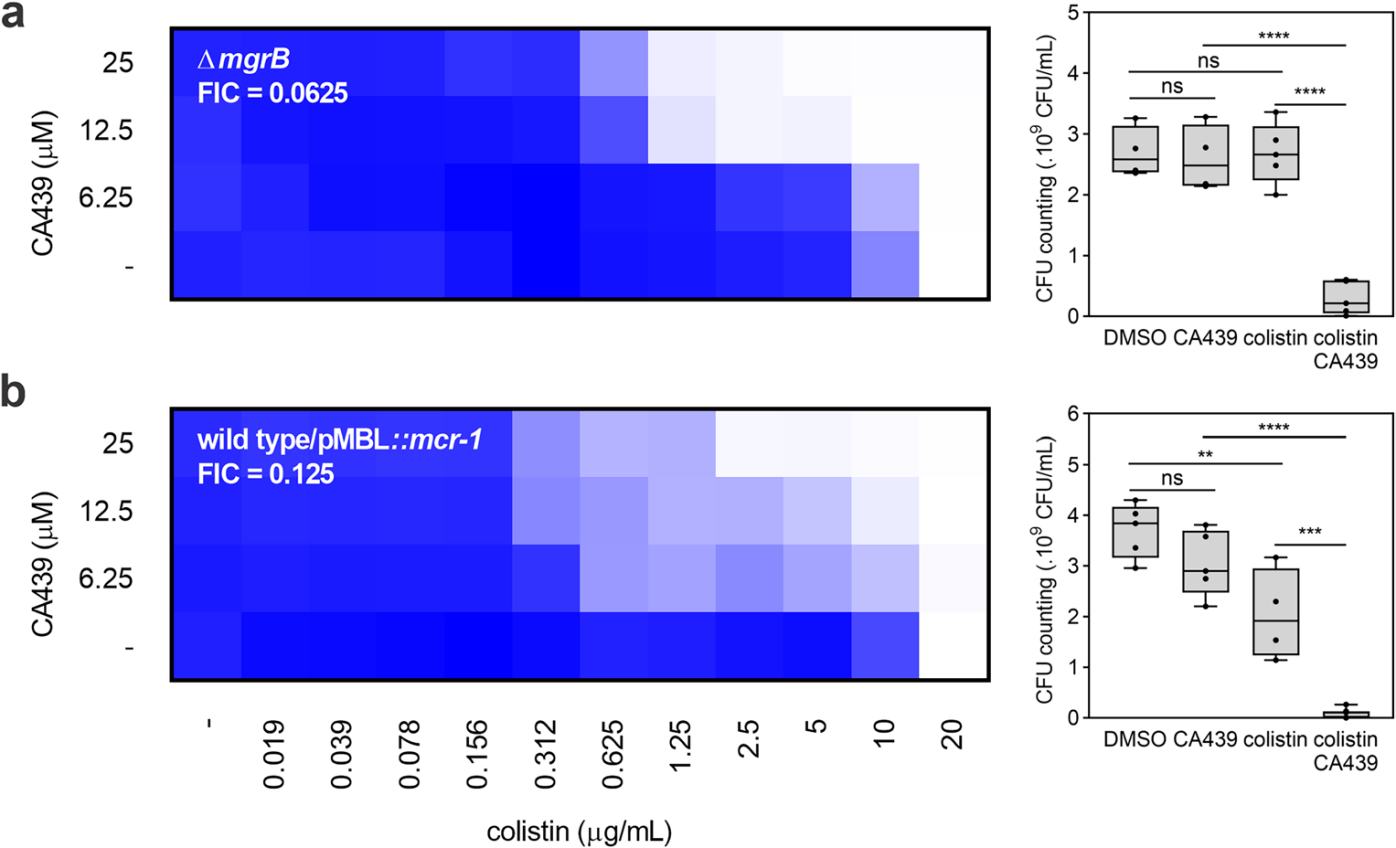
CA439 is effective against highly resistant colistin strains. Checkerboard assay showing CA439-colistin combination in (**a**) a *mgrB* mutant or (**b**) in a wild type strain overexpressing *mcr-1*. Dark blue regions represent higher cell density. Data represent the mean OD (600 nm) of at least three biological replicates. Right panel shows the CFU counting obtained in each growth condition: DMSO (LB-medium with the addition of DMSO), CA439 (25 μM CA439 alone), colistin (1.25 μg mL^−1^ and 2.5 μg mL^−1^ colistin alone for Δ*mgrB* and *mcr-1* respectively) and CA439 + colistin (25 μM CA439 + 1.25 μg mL ^−1^ colistin for Δ*mgrB* or 2.5 μg mL^−1^ colistin for *mcr-1*). Fractional inhibitory concentration (FIC) was obtained by dividing the colistin MIC in the presence of CA439 (1.25 μg mL^−1^ for Δ*mgrB* and 2.5 μg mL^−1^ for *mcr-1*) by the colistin MIC in the absence of CA439 (20 μg mL^-1^ for both strains). Statistical analysis was performed using one-way ANOVA with Tukey’s correction multiple comparison test (****P < 0.0001; ***P < 0.001; **P < 0.01; ns, no significant differences).

Resistance to colistin mainly occurs by the *pmrC*, *ugd* and the *arn*-mediated modification of LPS. However, two main acquired resistance mechanisms to colistin in Enterobacteriaceae have been described. Mutations or disruptions of *mgrB*, which encodes for MgrB, a transmembrane peptide that inhibits the PhoP/PhoQ system, derepress the system and thus increase resistance to polymyxins^31^. The plasmid-mediated colistin resistance (Mcr) is easily transferred among bacteria and confers resistance to colistin-susceptible strains. *mcr-1* encodes for the most frequent phosphoethanolamine (PEtN) transferase enzyme variant identified in resistant clinical isolates. MCR-1 incorporates PEtN into lipid A similar to the *pmrC* gene product^32^. Therefore, CA439-colistin effect was analyzed in a Δ*mgrB* mutant strain and in a wild type strain that overexpresses *mcr-1*^32^ (Fig. 8). In either case, CA439 caused a reduction in the MIC of colistin. As expected, a Δ*phoPQ* Δ*mgrB* double mutant strain rendered an equivalent MIC for colistin and FIC value to the single Δ*phoPQ strain (*Supplementary Fig. S7).

We evaluated whether we could extend the use of CA439 to other *Salmonella* strains that are resistant to colistin (*S.* Typhimurium STM23 and *S.* Enteritidis PT4) and in other bacterial genera of clinical interest (*E. coli*, *A. baumannii* and *K. aerogenes*). Although both *Salmonella* strains show a fourfold increase in colistin resistance (MIC of 2.5 μg mL^−1^ for 14028 s compared to 20 μg mL^−1^ for STM23 and PT4), CA439 was able to potentiate colistin, reaching FIC values of 0.03125 and 0.0625 for STM23 and PT4, respectively (Supplementary Fig. S8). *E. coli* strains BW25113 and MG1655 (FIC of 0.25), *A. baumannii* (FIC of 0.125) and *K. aerogenes* (FIC of 0.5) also presented enhanced susceptibility to the combined drugs when compared with the effect exerted by colistin alone (Supplementary Fig. S9).

### CA439 potentiates colistin-mediated lysis

We then evaluated whether CA439 was capable of altering colistin-mediated lysis of *Salmonella*. Either the wild type or Δ*mgrB* strains were preincubated with CA439 for 3 h, followed by treatment with a lytical concentration of colistin and the optical density of the cultures was monitored. Preincubation with CA439 favored bacterial cell lysis promoted by colistin (black curves) while the addition of DMSO had no effect (Supplementary Fig. S10, black curves). To analyze whether preincubation with CA439 was required, the quinazoline was added individually or simultaneously with colistin at 3 h (Supplementary Fig. S10, blue curves). Either protocol showed equivalent effects (Supplementary Fig. S10), indicating that preincubation was not a prerequisite. These results reinforce the notion that CA439 aids in destabilizing the bacterial membrane (Fig. 7).

### Colistin-CA439 protective effect in the *Galleria mellonella* infection model

To examine if the CA439-colistin combination could be used as an efficient therapy in vivo, *G. mellonella* was chosen as a suitable infection model^33^. No toxic effects were observed below 5 mg kg^−1^ and 25 mg kg^−1^ of colistin and CA439, respectively (Supplementary Fig. S11, left and middle panel). Because the combination of drugs showed toxic effects up to 0.25 mg kg^−1^ (Supplementary Fig. S11, right panel), we used 0.125 mg kg^−1^ (0.0625 mg kg^−1^ of each compound). Larvae groups were infected with 1 × 10^7^ CFU/larva of *Salmonella*. After 2 h, larvae were treated with colistin or CA439 alone or with the combination of drugs and survival was monitored up to 72 h post-infection. The results indicate that while each individual drug did not show significant difference when compared to the injection of PBS (control) up to 72 h post-infection, the treatment with the CA439 and colistin combination of the drugs showed 30 and 33% increase in survival of the larvae at 48 and 72 h post-infection, respectively (Fig. S12). As expected, the Δ*phoP* strain used as control was strongly attenuated in its virulence ability when compared to the wild type strain (Fig. S12).

## Discussion

Colistin, a polymyxin chemotype drug, is currently used as a last-resort therapy for bacterial infections caused by multidrug-resistant pathogens, such as *Salmonella enterica*, *Acinetobacter baumannii* and *Pseudomonas aeruginosa*. However, colistin doses applied with effective antimicrobial action are associated with significant toxic adverse effects so its use is carefully considered^17,31^.

In this study, we aimed to find a novel potential therapeutic approach for the treatment of salmonellosis, taking into account the mechanisms that allow this pathogen to counteract the effect of colistin. One key player is the *Salmonella* PhoP/PhoQ TCS which, upon interaction with polymyxins upregulates the expression of a set of genes including those that mediate the attachment of aminoarabinose to lipid A and the modification of phosphate groups (particularly with phospho-ethanolamine) in the LPS core region^24^. Such alterations diminish the charge density associated with LPS, thereby decreasing the affinity of the cationic colistin molecule for the bacterial surface. To attain a reduction in the effective antibacterial colistin concentration, we combined this knowledge with our previous identification of quinazoline molecules which selectively inhibit PhoQ autophosphorylation by competition with ATP^16^. We demonstrate that CA439 quinazoline lowers the MIC of colistin with a fractional inhibitory concentration (FIC) of 0.125. We compared the phenotypes of the wild type with single and double mutants that inactivate the main PhoP/PhoQ-dependent genes responsible for CAMPs resistance, including *phoP*, *phoQ,* and PhoP-indirectly regulated- PmrA/PmrB-dependent genes, *pmrC* and the *arn* operon (*pmrHIJKLM*). In all cases, the reduction in the FIC values relied on the inhibitory effect of the quinazoline on the activity of the PhoP/PhoQ system as a master regulator of the cascade. However, the inactivation of none of the aforementioned genes, including either *phoP* or *phoQ*, was able to completely abrogate the enhancing action of the quinazoline.

We determined that an *ugd* (also named *pmrE*) mutant strain was eightfold more susceptible to colistin than the wild type one, indicating that this gene product confers a layer of resistance beyond other PhoP/PhoQ-dependent genes. The *ugd* gene encodes the UDP-glucose dehydrogenase enzyme that converts UDP-glucose in UDP-glucuronic acid, the precursor molecule of synthetic pathways that render extracellular polysaccharides (including CA), and L-Ara4N, both factors required to counteract polymyxin deleterious action^24^.

*ugd* transcription depends on the concert of PhoP, PmrA and Rcs regulators, which directly bind to *ugd* promoter region, being PhoP required to activate *ugd* expression when bacteria are under a low magnesium, low Fe^3+^ concentration environment (in which PmrA is activated via the PhoP-PmrD cascade)^12^. In this condition, a double *pmrC* (deficient in PEtN modification in LPS) and *ugd* mutant strain (deficient in exopolysaccharides and L-Ara4N synthesis) showed a reduction in colistin MIC value by 16-fold when compared to the wild type one. Intriguingly, the single inactivation of main capsule synthetic genes, *wcaL*, *wzxc*, *wzb* and *wcaJ*, all known to be under Rcs regulation^28^, did not significantly alter neither colistin MIC levels nor the combined quinazoline-colistin effect. Therefore, the Rcs-dependent gene that plays a key role in the contribution to colistin resistance is *ugd*, mainly by its involvement in the synthesis of the UDP-glucuronic acid, the precursor of the pathway that renders L-Ara4N-modified LPS. Still, in this context, when the major described pathways that confer resistance cannot be activated (i.e., in the *phoP rcsB* double mutant strain), a remnant potentiation of colistin action by the quinazoline could be observed.

The quinazoline CA439 was unable to enhance the action of antibiotics that target bacterial pathways that differ from colistin, such as DNA transcription, RNA translation or cell wall peptidoglycan synthesis. These results indicate that the depolarization action of CA439 is not enough to exert a potentiating effect when combined with other antimicrobial agents distinct from colistin. Moreover, the quinazoline did not enhance the sub-lytic bacterial permeabilizing action of EDTA (a cation chelating drug) or SDS (anionic detergent). However, in addition to the inhibitory action over the PhoQ regulon, CA439 was effective in disrupting *Salmonella* OM electrochemical potential, that together with the action of colistin produces the collapse of its homeostatic mechanisms. Whilst it is well described that once polymyxins traverse the OM, they permeabilize the IM, which is required for bacterial lysis^34^, the mechanistic details of IM disruption have not been entirely elucidated. Recent work indicates that, once colistin reaches the periplasm, it can bind a pool of precursor LPS molecules that are associated to the IM and waiting to be transported to the outer membrane leaflet, and that the induced accumulation of LPS in the IM augments the bacterial susceptibility to colistin^19^. The fact that CA439 does not alter the IM integrity was also strengthen by its incapacity to enable the access of ONPG to be hydrolyzed by the cytoplasmic β-galactosidase enzyme. In contrast, and reinforcing the electrochemical imbalance results, only when it acts in conjunction with colistin, the quinazoline allows the entry of nitrocefin to be hydrolyzed by periplasmic β-lactamase. So, it is tempting to postulate that the electrochemical OM disturbance caused by the quinazoline facilitates the intrinsic capacity of colistin to disorganize the OM LPS, an effect that subsequently facilitates a better access to perturb the integrity of the cytoplasmic membrane. We also show that the dual drug treatment improved the lytic performance of colistin when compared treatment with an equivalent, sub-lytic concentration of colistin alone (the cartoon in Fig. S13 depicts our model for the concerted action of CA439).

One major drawback for the success of colistin antibacterial therapy, in addition to toxic liabilities over the host, is the evolving bacterial resistance due to acquired mutations in key components of the PhoP/PhoQ regulon^17,35,36^. Among PhoQ-regulating proteins, MgrB is the most widely distributed among Enterobacteriaceae^37^ Being a repressor of PhoQ, *mgrB* loss of function mutants exacerbate PhoQ-dependent resistance against colistin. In *Klebsiella pneumoniae* or in *Pseudomonas aeruginosa*, mutations or disruption of the *mgrB* gene have been shown to be prevalent among other chromosomal mutations in colistin-resistant clinical strains^38^. When CA439 is added, a *Salmonella mgrB* mutant showed a 16-fold decrease in colistin MIC values, pointing at the efficacy of the dual action of the compounds even when the PhoP/PhoQ system is derepressed. *S.* Typhimurium STM23 and *S.* enteritidis PT4 clinical isolates^39,40^ showed 16 to 32-fold increase in susceptibility to colistin when they were under the dual drug effect, demonstrating that the potentiating effect can be applied to strains with clinical impact, other than *S. enterica* serovar Typhimurium 14028 s.

A *Salmonella* strain which expresses *mcr-1*, one of the most extensively characterized and widespread mobilizable plasmid-borne resistance genes, assists in dissemination of colistin resistance to other pathogenic bacteria by horizontal transfer^32^, displayed eightfold reduction of the resistance to colistin when exposed to the combined treatment. In this case, the capacity of the *mcr-1* encoded enzyme in modifying LPS with PEtN residues would be insufficient to confer resistance when PhoP/PhoQ- and Rcs-dependent determinants for resistance have been inhibited by the quinazoline action. The combined antibacterial treatment was also more effective in *E. coli, A. baumannii* and *Klebsiella aerogenes* strains when compared with colistin alone. This correlates with the impact that conserved PhoP/PhoQ-PmrA/PmrB and Rcs-dependent mechanisms have in polymyxin resistance in these Gram-negative pathogens and this would extend the prospective application of the mixed treatment.

The clinical reintroduction of colistin, combined with the extensive use for veterinary medicine, increases the prevalence of resistant strains in livestock and the concomitant risk of animal-human transmission^17^. In this context, our results foresee an auspicious therapeutic outcome for the dual agent strategy when applied to treat highly resistant, clinically hazardous strains.

The combination of colistin and quinazoline mitigated *Salmonella*’s lethal effects in a systemic infection model using *Galleria mellonella* wax moth larvae when compared to the administered sub-lethal concentration of each single-drug. Overall, our results encourage further pursuit of the therapeutic implementation of the combined quinazoline-colistin treatment in mammalian hosts to combat *Salmonella* infections.

## Materials and methods

### Bacterial strains, cell culture and growth conditions

A list of the strains and plasmids used in this work is provided in Supplementary Table S1. Bacteria were routinely grown in Miller’s Luria–Bertani (LB) medium or on LB agar plates at 37 °C supplemented with appropriated antibiotics (kanamycin, 50 μg mL^−1^; chloramphenicol, 20 μg mL^−1^; ampicillin, 100 μg mL^−1^; tetracycline, 12.5 μg mL^−1^; spectinomycin, 50 μg mL^−1^).

Strains carrying gene deletions or chromosomal *lacZ* reporter fusions were generated by Lamba Red-mediated recombination followed by P22-mediated transduction using previously described protocols^41^. Insertion mutations in *pmrA*, *arnA* and *pmrC* were constructed with the one-step chromosomal inactivation method^42^. The promote regions of *arn* and *pmrCAB* operons were amplified by PCR and ligated into the pPROBE(NT) plasmid^43^. Primers used are indicated in Supplementary Table S2.

All reagents and chemicals were from Sigma, except the Luria–Bertani culture medium that was from Difco. Colistin was obtained from Sigma (Colistin sulfate salt, Cat#4461). Oligonucleotides and enzymes were purchased from Life Technologies. SMILES and Labbook codes for the compounds are provided in Supplementary Table S3 and they were synthesized as previously described^16^.

### Checkerboard Assay

Overnight cultures (18 h) of each strain grown in LB medium were sub-cultured 1:10.000 in fresh LB medium supplemented with CA439 (or CA439-analogues) and added to a 96-well assay plate containing the indicated twofold dilutions of colistin (EDTA or SDS when appropriate). Bacteria were grown at 37 °C with shaking at 250 rpm to stationary phase and OD_600_ was measured using a Synergy 2 plate reader. OD_600_ data was converted to percent growth and heatmaps were generated in GraphPad (version 6.01; GraphPad Software, San Diego, CA, USA), with a scale between 0–100% growth in which dark blue regions represent higher cell density and light blue regions lower cell density. Raw data for each strain assayed is provided in Supplementary Table S4. The MIC values were determined to be the condition that resulted in a percent residual growth of ≤ 10%. The FIC was determined to be the MIC of colistin combined with the indicated concentration of the compound divided by the MIC of colistin alone. Overnight cultures were used for serial dilutions to determine the colony-forming units (CFU) in each condition. CFU evaluations were carried out with individual sub-inhibitory concentrations of CA439 or of colistin, or with the combination of these concentrations of drugs, as indicated in the legend of the corresponding figures.

### β-galactosidase activity assays

Bacteria were grown overnight with shaking at 37 °C in LB media with the addition of CA439, CA490, colistin or colistin combined with CA439 or CA490 at the final concentration indicated in each experiment. Kanamycin (50 μg mL^−1^) or chloramphenicol (20 μg mL^−1^) were added when required. β-galactosidase activity was determined as described^44^. Relative activity was calculated taking the activity obtained in DMSO as 100%.

### GFP fluorescence assays

The *S.* Typhimurium wild type strain carrying the pP*arn-gfp* or pP*pmrCAB-gfp* reporter plasmid or Δ*phoP* and Δ*phoPQ* strains carrying the pP*pmrCAB-gfp* reporter plasmid were grown with shaking overnight at 37 °C. Cultures were subcultured 1:100 in LB medium and supplemented with FeCl_3_ (100 μM), MgCl_2_ (5 mM) or CA439 (25 μM) for the wild type strain or with the indicated concentration of FeCl_3_ and CA439 (25 μM) or the equivalent of DMSO for Δ*phoP* and Δ*phoPQ* strains and were incubated at 37 °C with shaking for 16 h. Optical density at 600 nm (OD_600_) and GFP fluorescence (excitation at 485/emission at 528 nm) were determined in a microwell plate reader (Synergy 2). Transcriptional activity was calculated as the ratio of GFP fluorescence and OD_600_ (FU/OD_600_). Relative FU/DO_600_ was calculated by taking the activity obtained in DMSO as 100%.

### *DiSC*_*3(5)*_* assay*

Cells were grown to early-exponential phase in LB media supplemented with 5 mM EDTA to disrupt the outer membrane and promote DiSC_3(5)_ access to the cytoplasmic membrane. Cells were harvested, washed twice with 5 mM Bis–Tris/Trizma, 20 mM glucose pH 7, and resuspended in the same buffer to a final OD_600_ of 0.1 with 1 μM DiSC_3(5)_. Cells were transferred to a gently stirred cuvette at 37 °C and DiSC_3(5)_ fluorescence (excitation = 620 nm, emission = 670 nm) was read immediately after using a Cary Eclipse Fluorescence Spectrophotometer (Agilent). After steady-state fluorescence was reached (5 min), 5 μg mL^-1^ colistin was added (5 min) and then the indicated concentration of CA439 (CA490 or the equivalent of DMSO) was added. The fluorescence intensity was recorded for at least 15 min after compound addition.

### Outer membrane integrity assay

The β-lactamase assay was performed as previously described^45,46^. *S.* Typhimurium 14028 s pBR322 cells grown overnight in LB medium with 100 μg mL^−1^ ampicillin were subcultured 1:50 in fresh LB with 50 μg mL^−1^ ampicillin and grown at 37 °C to early-exponential phase. Cells were harvested, washed in PBS and resuspended to OD_600_ = 0.02. A volume of 50 μL of the cell suspension was added to a 96-well plate containing 50 μL of PBS with a final concentration of 30 μM nitrocefin and the antibiotic, the compound or the combination indicated in each assay. Plates were incubated at 37 °C and absorbance at 492 nm was read after 2 h using a microwell plate reader (Synergy 2) to monitor nitrocefin hydrolysis.

### Inner Membrane Integrity Assay

The β-galactosidase assay was performed as previously described^45,46^ .*S.* Typhimurium 14028 s *cpxP::lacZ* cells grown overnight in LB medium, subcultured 1:50 in fresh LB and grown at 37 °C to early-exponential phase. Cells were harvested, washed in PBS and resuspended to OD_600_ = 0.4. A volume of 50 μL of the cell suspension was added to a 96-well plate containing 50 μL of PBS with a final concentration of 2 mM *o*-nitrophenyl-*β*-galactoside (ONPG) and the antibiotic, the compound or the combination indicated in each assay. Plates were incubated at 37 °C and absorbance at 405 nm was read after 1 h using a microwell plate reader (Synergy 2) to monitor ONPG hydrolysis.

### Colistin-mediated lysis curves

Overnight cultures (18 h) grown in LB medium were subcultured 1:100 in fresh LB medium supplemented with CA439, CA490 or the equivalent of DMSO (black curves). After 3 h, a concentration of colistin equivalent to 6xMIC (for wild type) or 1xMIC (for Δ*mgrB*) was added alone or combined with CA439 or CA490. Bacteria were grown at 37 °C with shaking at 200 rpm and OD_600_ was measured every hour. After 8 h, serial dilutions of the cultures were made for colony-forming units’ determination.

### G. mellonella infection model

*G. mellonella* survival assays were performed as described previously^47,48^. Briefly, 16 larvae per group were selected by their weight, variable between 0.18 and 0.3 g, and incubated overnight at 37 °C without food. Each group was inoculated with bacterial suspensions prepared in PBS buffer (saline) of the strains under study at the CFU/larva values mentioned in the text. The inoculum was prepared at a fixed concentration, determined by plate count, in a PBS buffer and 10% glycerol, frozen and then diluted to obtain the necessary concentration to inoculate^49^. The chosen inoculation site was the last left proleg. The compounds analyzed were administered to the right proleg 2 h after inoculation with the bacteria. For the survival curves, the status of the larvae was checked every 24 up to 72 h post-infection.

### Statistical analysis

To test for statistical differences between means, one-way analysis of variance (ANOVA) and the Tukey multiple comparison test with an overall significance level of 0.05 were used. Calculations were performed with GraphPad Prism statistical software. When required, unpaired *t* test was used with the same overall significance level. Kaplan–Meier curves were constructed to analyze the toxic effect of the compounds over *Galleria* larvae^50^, while two-way ANOVA statistical analysis was used to analyze the *Galleria* larvae survival assay.

### Supplementary Information

Supplementary Information 1.

Supplementary Information 2.

## Data Availability

The datasets used and/or analysed during the current study are available from the corresponding author on reasonable request.
