# Supplementary material for: Enhancing colistin efficacy against Salmonella infections with a quinazoline-based dual therapeutic strategy
Source: Sci Rep. 2024 Mar 1;14:5148. doi: 10.1038/s41598-024-55793-0 (PMC10907601; doi:10.1038/s41598-024-55793-0)
Supplement: Supplementary file 1 — Supplementary Information 1. [file 41598_2024_55793_MOESM1_ESM.docx]

**Enhancing Colistin Efficacy Against *Salmonella* Infections with a Quinazoline-Based Dual Therapeutic Strategy**

**Supplementary Information**

**Supplementary Figure and Legends**

**
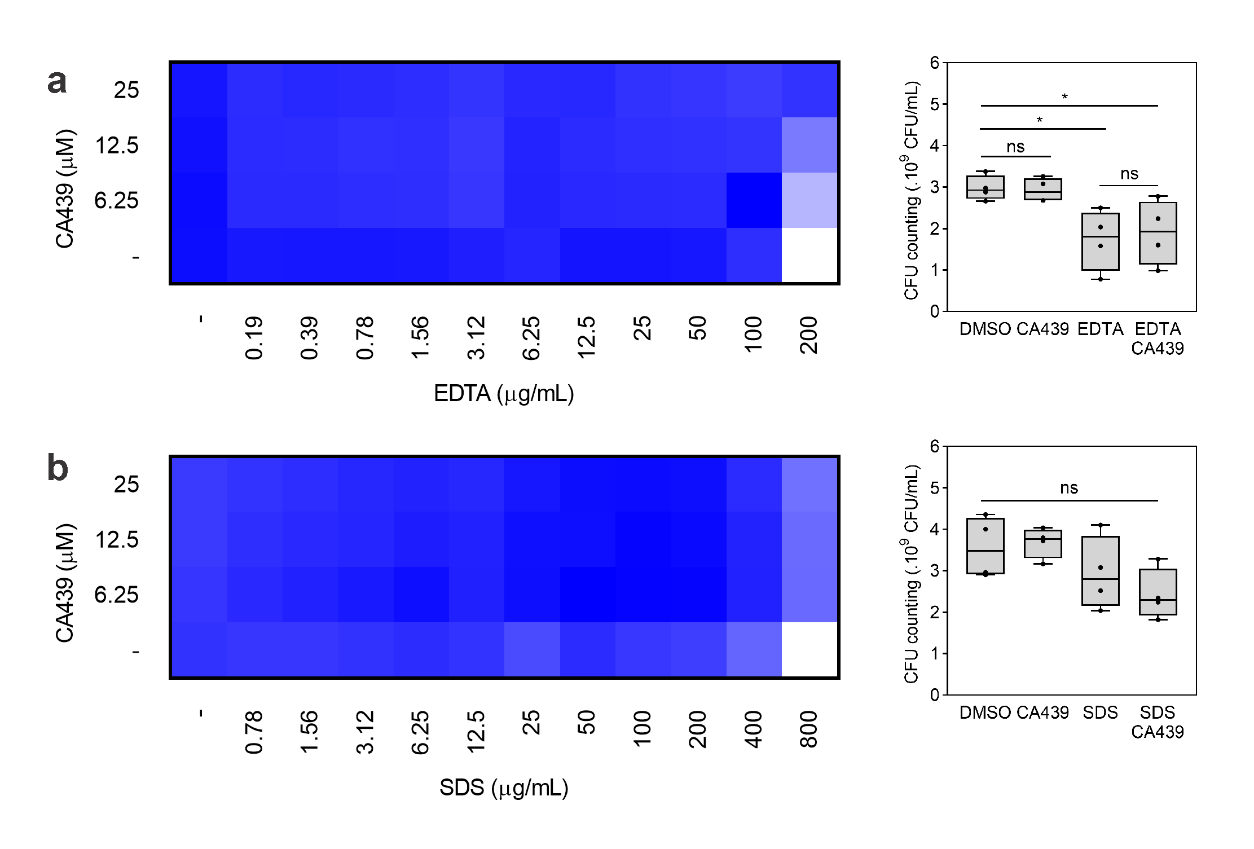
**

**Figure S1. Effect of permeabilizing agents on CA439-colistin action.** Checkerboard assay showing coincubation of CA439 with increasing concentrations of **a**, EDTA or **b**, SDS. Dark blue regions represent higher cell density. Data represent the mean OD (600 nm) of at least three biological replicates. Right panel shows the CFU counting obtained in each growth condition: DMSO (LB-medium with the addition of DMSO), CA439 (25 μM CA439 alone), EDTA (100 μg mL^-1^) or SDS (400 μg mL^-1^) alone and CA439 + EDTA or SDS (25 μM CA439 + 100 μg mL^-1^ or 400 μg mL^-1^, respectively). Statistical analysis was performed using one-way ANOVA with Tukey’s correction multiple comparison test (** P* < 0.01; ns, no significant differences).


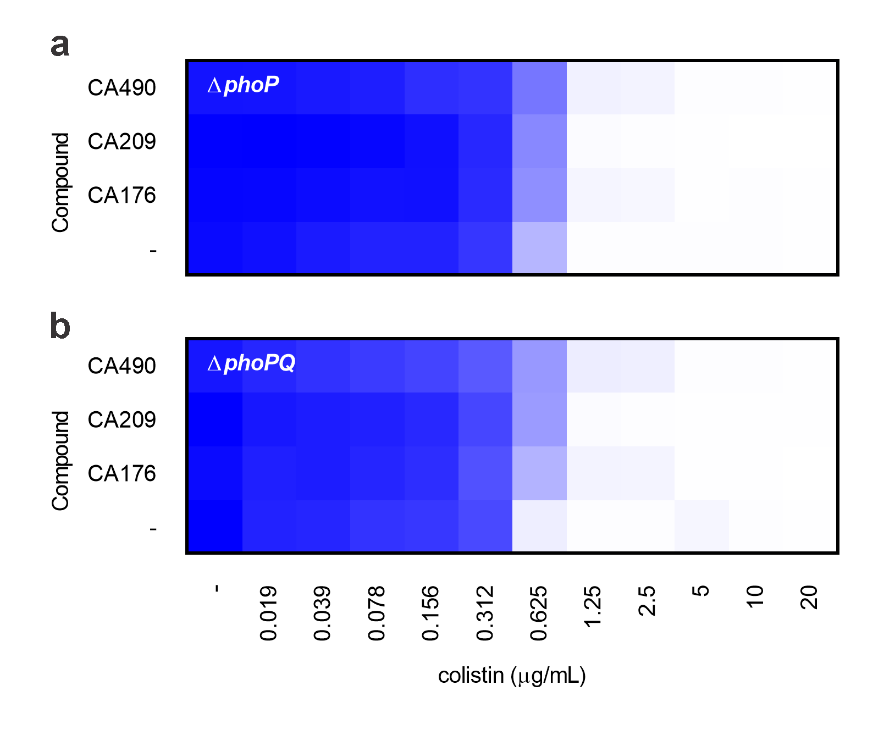


**Figure S2. PhoP/PhoQ-independent mechanism of CA439-colistin potentiation is also specific to its chemical structure.** Checkerboard assay showing co-incubation of colistin with 25 μM CA-analogues (CA176, CA209, CA490) in **a**, *phoP* mutant or **b**, *phoPQ* mutant. Dark blue regions represent higher cell density. Data represent the mean OD (600 nm) of at least three biological replicates.


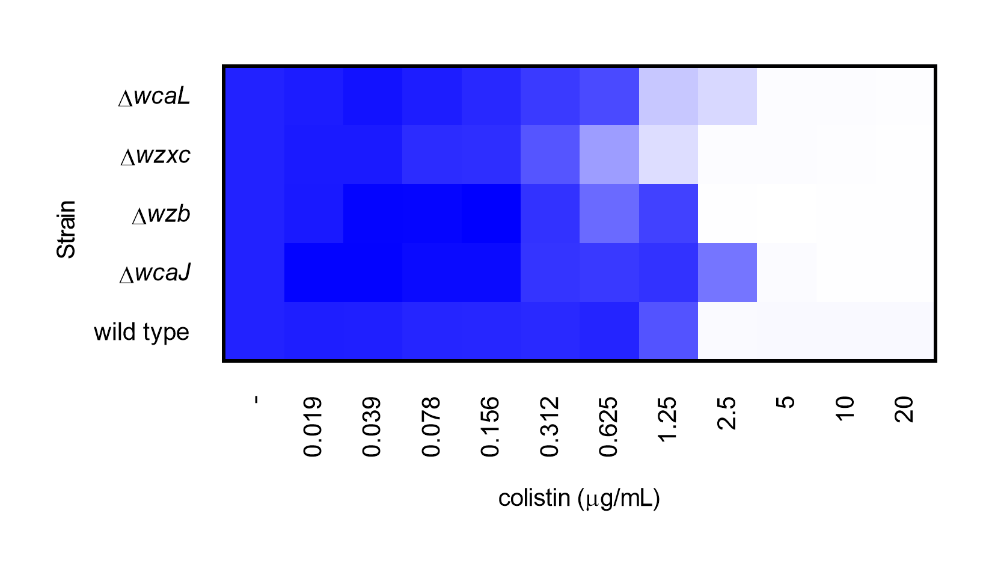


**Figure S3. Colanic acid production does not confer resistance to colistin.** Susceptibility to increasing concentrations of colistin in the wild type strain and single mutant strains in colanic acid synthesis genes (Δ*wcaJ*, Δ*wzb*, Δ*wzxc* or Δ*wcaL*). Dark blue regions represent higher cell density. Data represent the mean OD (600 nm) of at least three biological replicates.


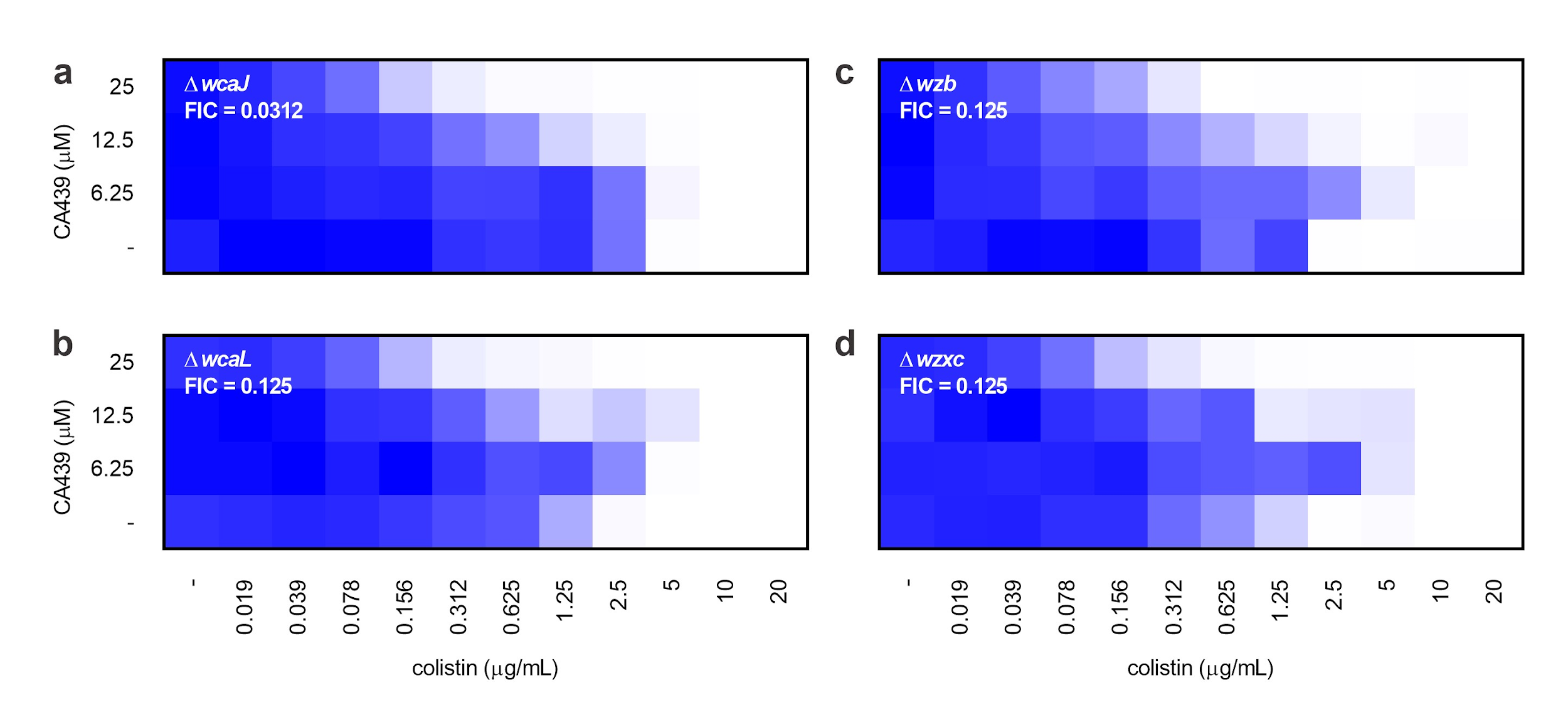


**Figure S4. CA439-colistin potentiation is independent of colanic acid synthesis genes.** Checkerboard assay showing potentiation of colistin by CA439 in **a**, *wcaJ*, **b**, *wcaL*, **c**, *wzb* and **d**, *wzxc* mutants. Dark blue regions represent higher cell density. Data represent the mean OD (600 nm) of at least three biological replicates. Fractional inhibitory concentration (FIC) was obtained by dividing the colistin MIC in the presence of CA439 (0.156 μg mL^-1^ for Δ*wcaJ* and 0.312 μg mL^-1^ for Δ*wzb*, Δ*wcaL* and Δ*wzxc*) by the colistin MIC in the absence of CA439 (5 μg mL^-1^ for Δ*wcaJ* and 2.5 μg mL^-1^ for Δ*wzb*, Δ*wcaL* and Δ*wzxc*).


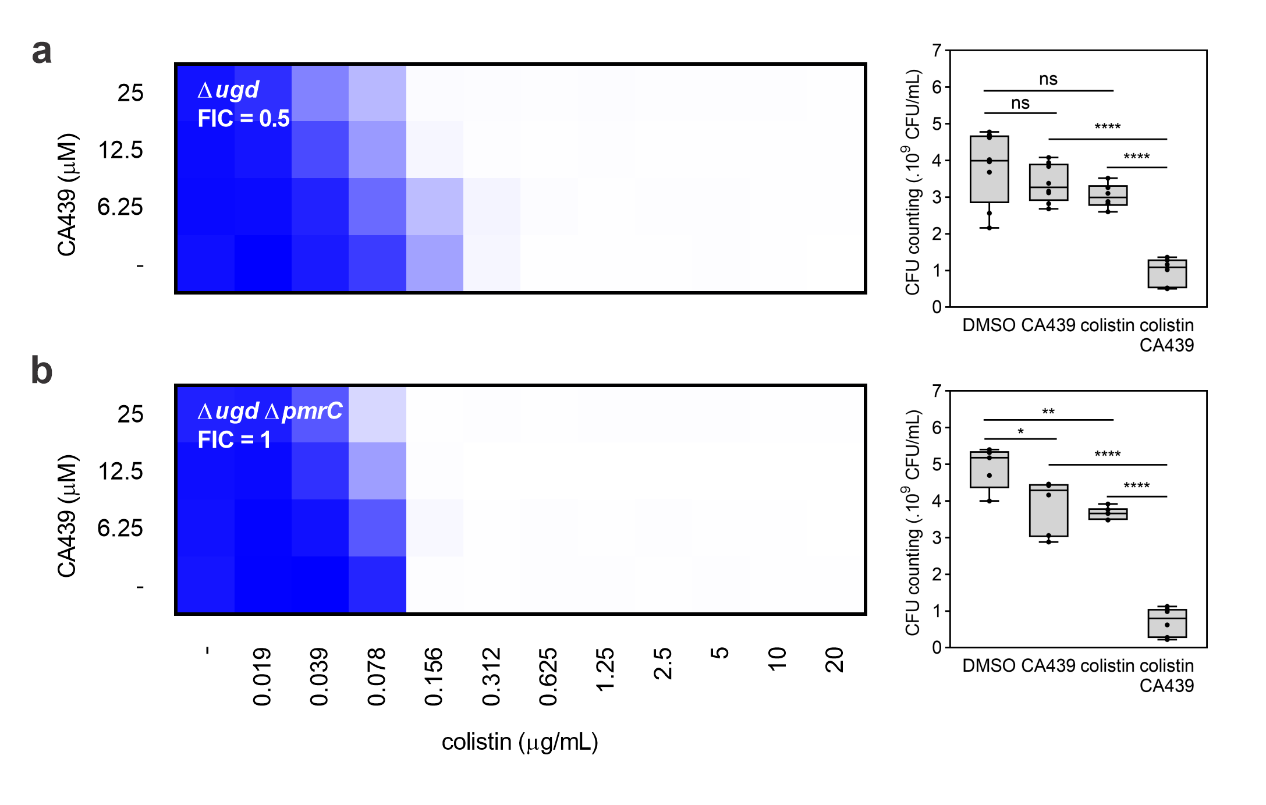


**Figure S5. CA439-colistin potentiation relieves in a Δ*ugd* Δ*pmrC* background.** Checkerboard assay showing CA439-colistin combination in **a**, *ugd* mutant or **b**, *ugd pmrC* double mutant. Dark blue regions represent higher cell density. Data represent the mean OD (600 nm) of at least three biological replicates. Right panel shows the CFU counting obtained in each growth condition: DMSO (LB-medium with the addition of DMSO), CA439 (25 μM CA439 alone), colistin (0.156 μg mL^-1^ for Δ*ugd* and 0.078 μg mL^-1^ for Δ*ugd* Δ*pmrC*) and CA439 + colistin (25 μM CA439 + 0.156 μg mL^-1^ colistin for Δ*ugd* and 0.078 μg mL^-1^ colistin for Δ*ugd* Δ*pmrC*). Fractional inhibitory concentration (FIC) for Δ*ugd* strain was obtained by dividing the colistin MIC in the presence of CA439 (0.156 μg mL^-1^ for both strains) by the colistin MIC in the absence of CA439 (0.312 μg mL^-1^ for Δ*ugd* and 0.156 μg mL^-1^ for Δ*ugd* Δ*pmrC* strain). Statistical analysis was performed using one-way ANOVA with Tukey’s correction multiple comparison test (**** *P* < 0.0001; ** *P* < 0.001; * *P* < 0.01; ns, no significant differences).


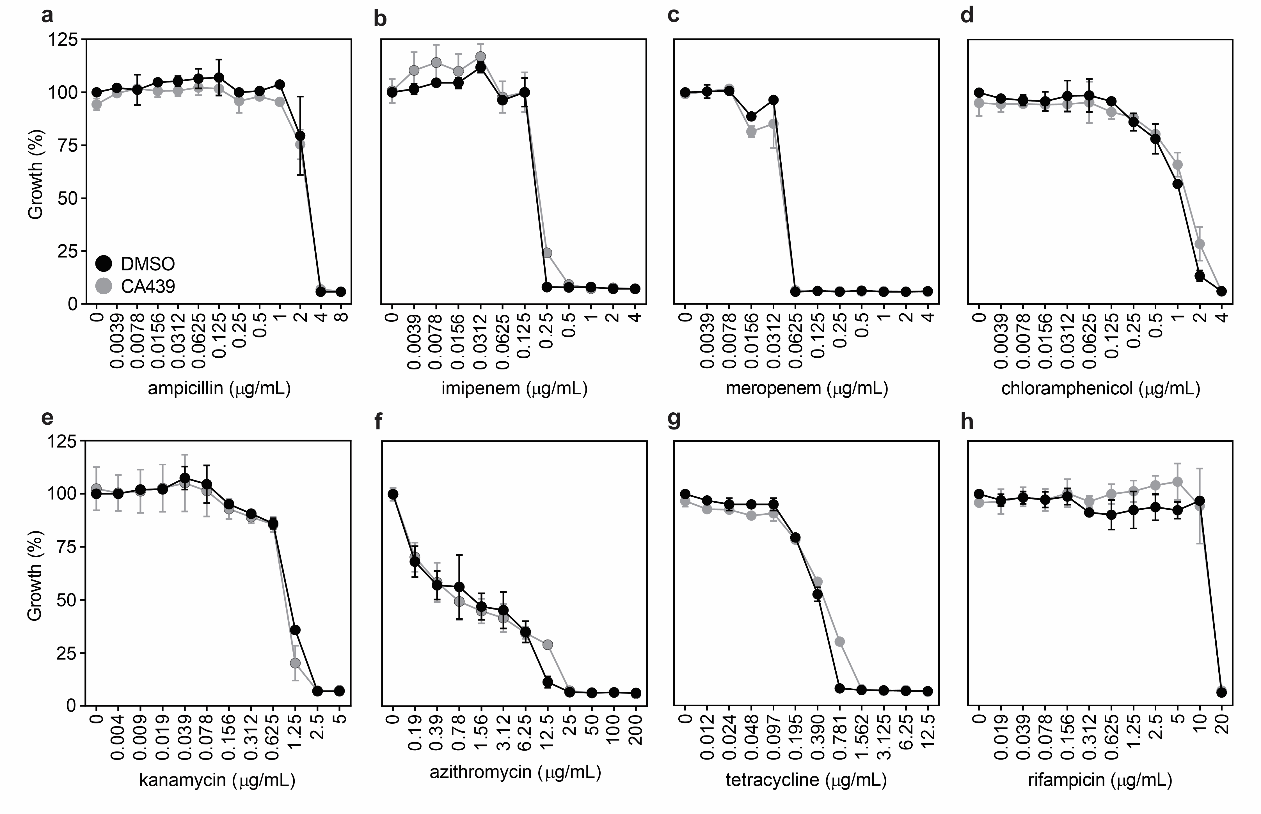


**Figure S6. CA439 selectively potentiates polymyxins.** Combinatorial analysis of subinhibitory concentrations of peptidoglycan-acting (ampicillin, imipenem or meropenem), protein translation-acting (kanamycin, chloramphenicol, tetracycline or azithromycin) or a gene transcription-acting antibiotic (rifampicin) in the presence (gray curve) or in the absence (black curve) of 25 μM CA439. OD (600 nm) was measured for each condition and relative growth was obtained using LB-medium with the addition of DMSO as 100%. Results represent the average of at least three biological replicates.


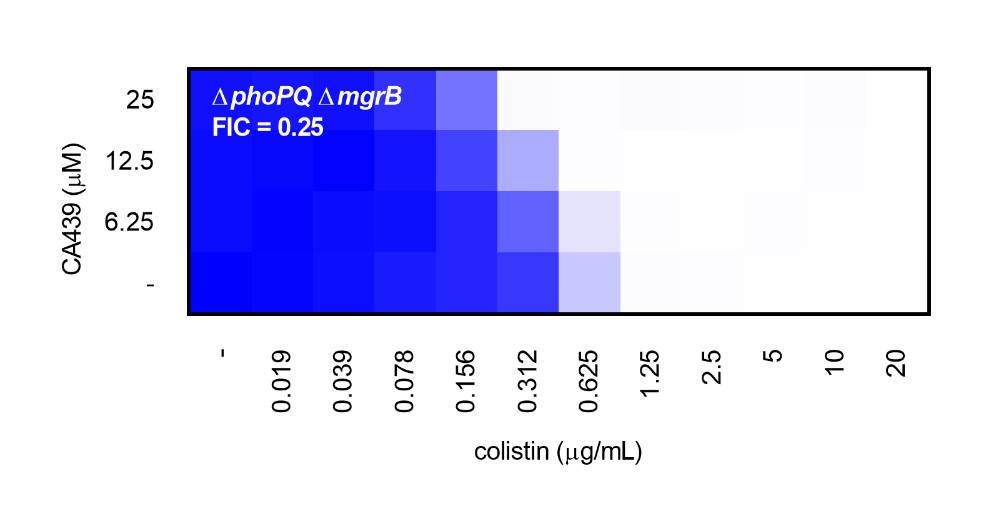


**Figure S7. Colistin-CA439 combination in a Δ*phoPQ* Δ*mgrB* background.** Checkerboard assay showing CA439-colistin combination in Δ*phoPQ* Δ*mgrB* double mutant. Dark blue regions represent higher cell density. Fractional inhibitory concentration (FIC) was obtained by dividing the colistin MIC in the presence of CA439 (0.312 μg mL^-1^) by the colistin MIC in the absence of CA439 (1.25 μg mL^-1^). Data represent the mean OD (600 nm) of at least three biological replicates.


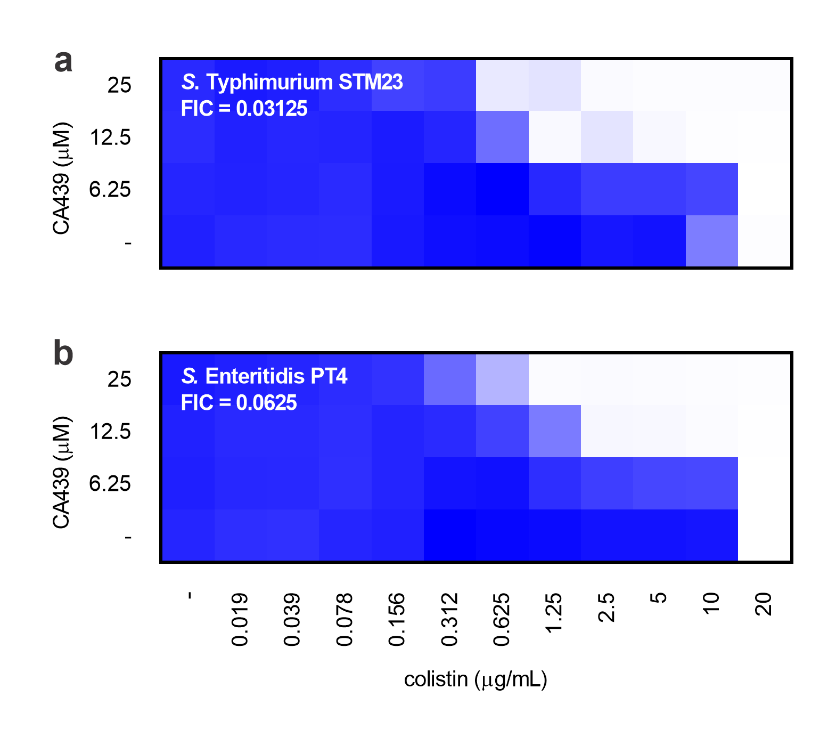


**Figure S8. CA439 is effective against other high-resistant *Salmonella* strains.** Checkerboard assay showing CA439-colistin combination in **a**, *S.* Typhimurium STM23 or **b**, *S.* Enteritidis PT4. Dark blue regions represent higher cell density. Fractional inhibitory concentration (FIC) was obtained by dividing the colistin MIC in the presence of CA439 (0.625 μg mL^-1^ for STM23 and 1.25 μg mL^-1^ for PT4) by the colistin MIC in the absence of CA439 (20 μg mL^-1^ for both strains). Data represent the mean OD (600 nm) of at least three biological replicates.


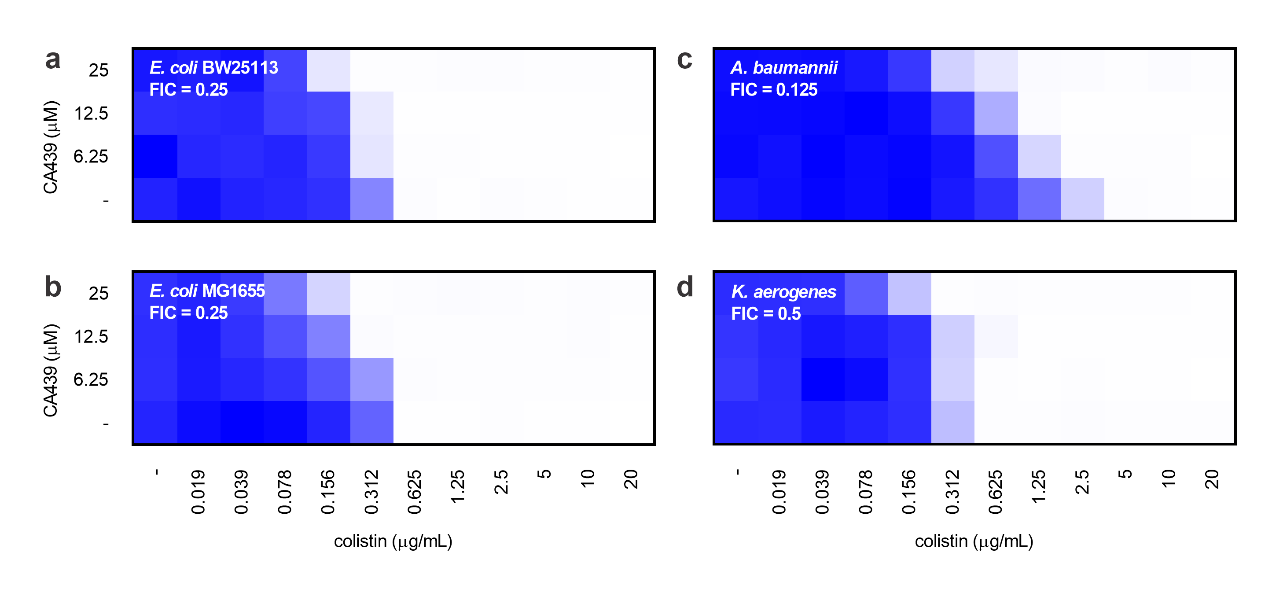


**Figure S9. CA439-colistin combination in other bacterial genera.** Checkerboard assay in the presence of CA439-colistin combination in **a,** *E. coli* BW25113, **b,** *E. coli* MG1655, **c,** *A. baumannii* ATCC 17978 and **d,** *K. aerogenes.* Dark blue regions represent higher cell density. Fractional inhibitory concentration (FIC) was obtained by dividing the colistin MIC in the presence of CA439 (0.156 μg mL^-1^ for *E. coli* strains, 0.312 μg mL^-1^ for *A. baumannii* and *K. aerogenes*) by the colistin MIC in the absence of CA439 (0.625 μg mL^-1^ for *E. coli* strains and *K. aerogenes*, 2.5 μg mL^-1^ for *A. baumannii*). Data represent the mean OD (600 nm) of at least three biological replicates.


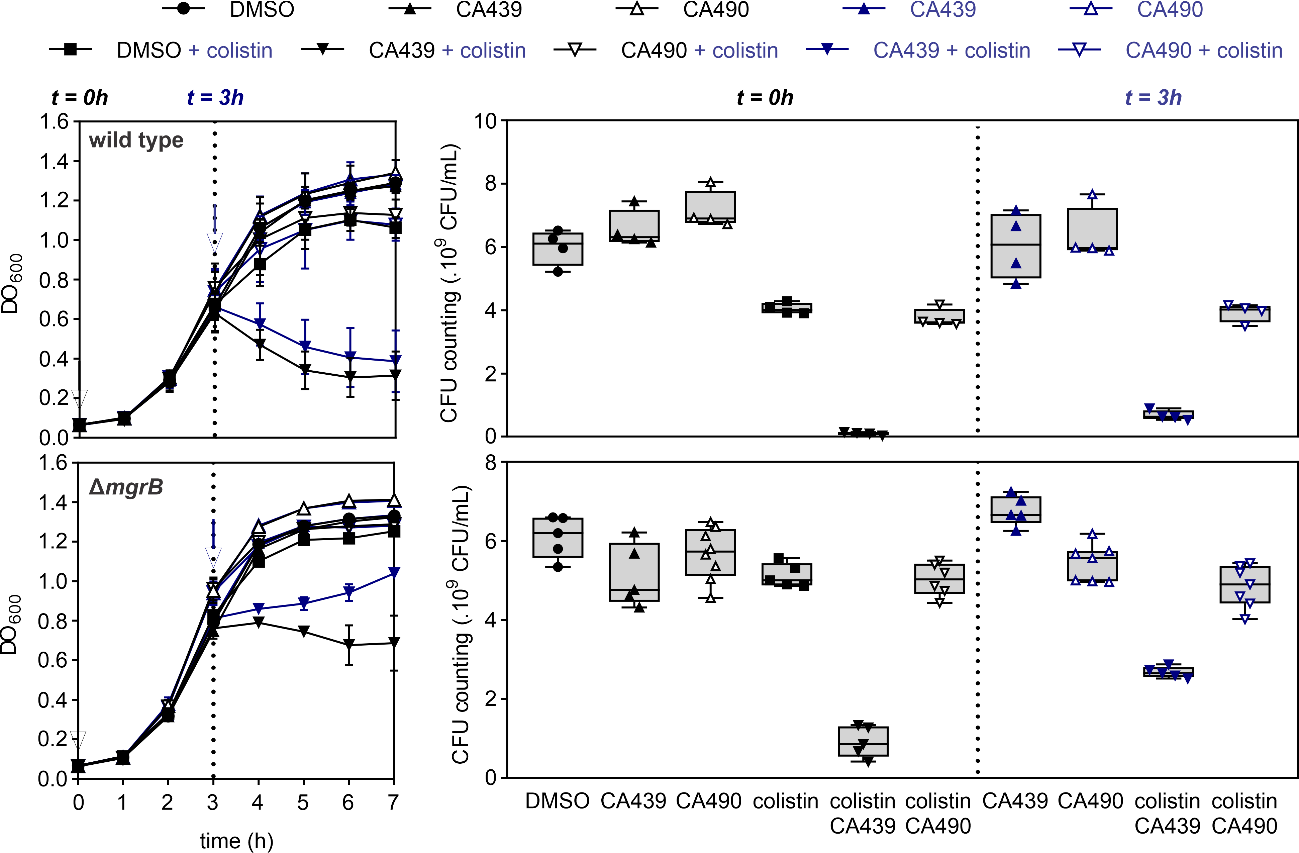


**Figure S10. CA439 potentiates colistin-mediated lysis.** Wild type (top panel) and Δ*mgrB* (bottom panel) strains were incubated with CA439, CA490 (25 μM) or DMSO for 3 hours and colistin was added (indicated by a blue arrow; 15 μg mL^-1^ for wild type and 20 μg mL^-1^ for Δ*mgrB*) and the optical density was monitored for 7 hours (black curves). Blue curves correspond to cultures in which the addition of the individual compound (CA439 or CA490) or combined with colistin was carried out at 3 hours. The results represent the average of three independent experiments. Right panel shows the CFU counting obtained in each growth condition.


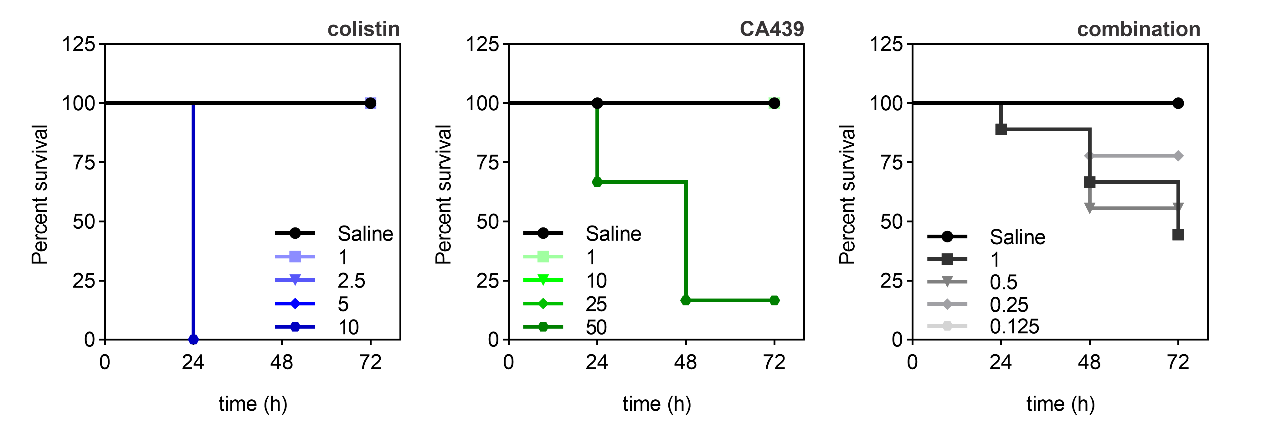


**Figure S11. Toxicity effect of colistin, CA439 or the combination over *G. mellonella* larvae.** Kaplan-Meier survival plots of *G. mellonella* upon injection with the indicated concentration of colistin (left panel), CA439 (middle panel) or the CA439-colistin combination (right panel). Survival assays were performed using at least 16 larvae in the saline group and 32 larvae in the other groups. Concentration values are expressed in mg kg^-1^. Combined conditions correspond to equal parts of each drug (e.g., 1 mg kg^-1^ in the right panel corresponds to 0.5 mg kg^-1^ of colistin + 0.5 mg kg^-1^ of CA439). Saline, saline solution. Percent survival values rendered overlapped lines in the graph corresponding to saline and 1, 2.5 and 5 mg kg^-1^ (left panel); to saline, 1, 10 and 25 mg kg^-1^ (middle panel); and saline and 0.125 mg kg^-1^ (right panel).


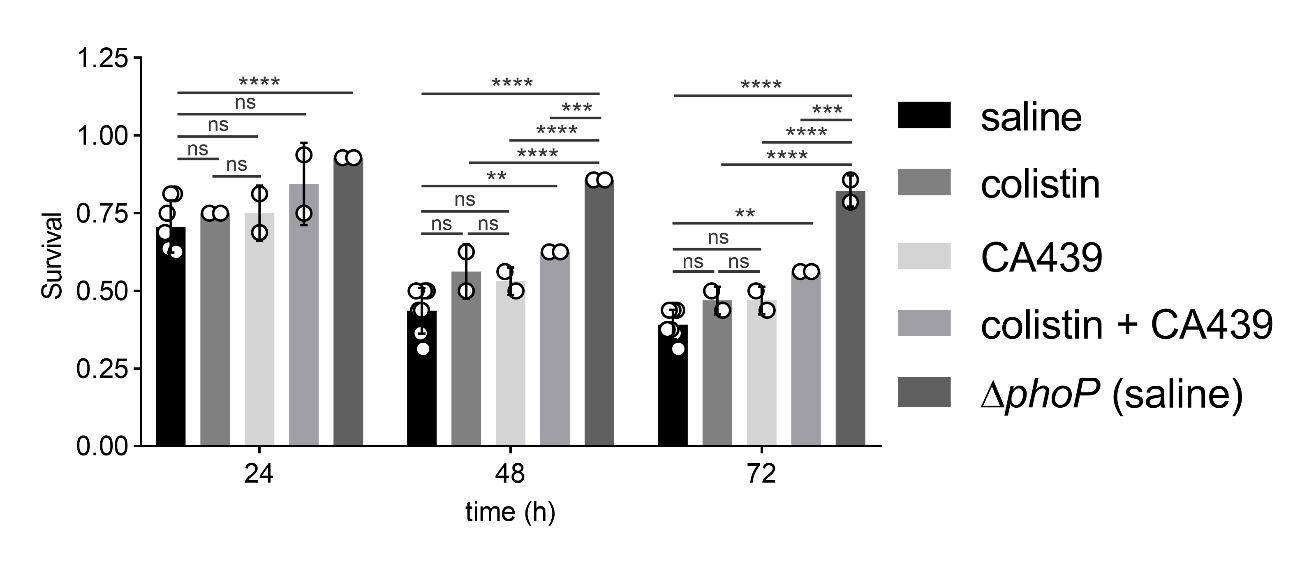


**Figure S12. CA439-colistin combination in a *G. mellonella* infection model.** Survival plot of *G. mellonella* upon injection with 1x10^7^ CFU/larva of *Salmonella* and treated with colistin (0.125 mg kg^-1^), CA439 (0.125 mg kg^-1^) or CA439-colistin combination (0.0625 mg kg^-1^ each) 2 h postinfection. Survival assays were performed using 32 larvae per group. Δ*phoP* strain (1x10^7^ CFU/larva) was used as control. Saline, saline solution. Statistical analysis was performed using two-way ANOVA with Sidak’s test for multiple comparisons (**** *P* < 0.0001; *** *P* < 0.0002; ** *P* < 0.0021; ns, no significant differences)


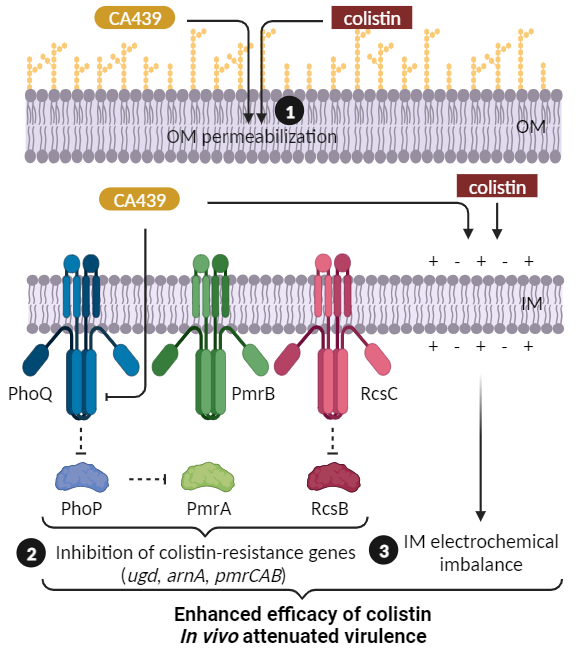


**Figure S13. Proposed model for CA439-colistin potentiation.** Quinazoline CA439 enhances the action of the antimicrobial peptide colistin through a triple mechanism of action that involves: 1, permeabilization of the bacterial outer membrane; 2, down-regulation of polymyxin resistance genes through the PhoP/PhoQ-PmrA/PmrB cascade; 3, alteration of electrochemical potential of the bacterial inner membrane.

**Supplementary Tables**

**Table S1. Strains and plasmids used in this study**

| **Strain or plasmid** | **Genotype and/or comments** | **Reference or source** | |
| --- | --- | --- | --- |
| ***S.* Typhimurium 14028s strains** | | | |
| 14028s | wild type | ATCC | |
| MS7953s | Δ*phoP::Tn10* | ^1^ | |
| PB11745 | Δ*phoQ::Tn10* | ^2^ | |
| PB2069 | Δ*phoPQ::Sp* | ^3^ | |
| PB15100 | Δ*pmrA::Cm* | This work | |
| PB15047 | Δ*phoP::Tn10* Δ*pmrA::Cm* | This work | |
| PB15139 | Δ*arnA::Km* | This work | |
| PB15140 | Δ*pmrC::Cm* | This work | |
| PB15141 | Δ*arnA::Km* Δ*pmrC::Cm* | This work | |
| PB3844 | *wcaJ::lacZ::Km* | Laboratory stock | |
| PB14361 | *wzb::lacZ::Km* | Laboratory stock | |
| PB9564 | *wcaJ::lacZ::Km* Δ*rcsB::Cm* | Laboratory stock | |
| PB15232 | *wzb::lacZ::Km* Δ*rcsB::Cm* | Laboratory stock | |
| PB14365 | *wcaL::lacZ::Km* | Laboratory stock | |
| PB14362 | *wzxc::lacZ::Km* | Laboratory stock | |
| PB7211 | Δ*rcsB::Cm* | Laboratory stock | |
| PB15294 | Δ*phoP::Tn10* Δ*rcsB::Cm* | This work | |
| PB2407 | *ugd::MudJ* | Laboratory stock | |
| PB15271 | *ugd::MudJ* Δ*pmrC::Cm* | This work | |
| PB11804 | Δ*mgrB::Cm* | ^4^ | |
| PB15042 | wild type/pMBL*::mcr-1* | This work | |
| PB15108 | wild type/pP*arn-gfp* | This work | |
| PB15109 | wild type/pP*pmrCAB-gfp* | This work | |
| PB15121 | Δ*phoP*/pP*pmrCAB-gfp* | This work | |
| PB15119 | Δ*phoPQ*/pP*pmrCAB-gfp* | This work | |
| PB10666 | *cpxP::lacZ::Cm* | Laboratory stock | |
| **Other *Salmonella* strains** | | | |
| PB12677 | *S.* Typhimurium STM23 | ^5^ | |
| PB12678 | *S.* Enteritidis PT4 | ^6^ | |
| **Other strains** | | | |
| PB12713 | *Acinetobacter baumannii* 17978 | | ATCC |
| PB10581 | *Klebsiella aerogenes* | | Laboratory stock |
| PB14288 | *Escherichia coli* MG1655 | | ATCC |
| PB11404 | *Escherichia coli* BW25113 | | ATCC |
| **Plasmids** | | | |
| pPROBE(NT) | Ori V, Km^R^ | ^7^ | |
| pKD3 | Ori R6K, Cm^R^, Amp^R^ | ^8^ | |
| pKD4 | Ori R6K, Km^R^, Amp^R^ | ^8^ | |
| pKD46 | ParaB-γ-β-exo, Ori R101, Amp^R^ | ^8^ | |
| pP*arn-gfp* | pPROBE(NT)::prom*arn* Km^R^ | This work | |
| pP*pmrCAB-gfp* | pPROBE(NT)::prom*pmrCAB* Km^R^ | This work | |
| pMBL::mcr-1 | pMBL*::mcr-1* | Laboratory stock | |

**Table S2. Cloning and Mutant Generation**

| **Primer** | **Sequence (5’-3’)*^a^*** | **Description** |
| --- | --- | --- |
| pmrA-FRT-Fw | agatactgattgttgaagacgacacgctattattacgtaggctggagctgcttcg | Δ*pmrA* strain |
| pmrA-FRT-Rv | ttagctttcctcagtggcaaccagcatgtagccaaaccgggaattagccatggtccatatg | Δ*pmrA* strain |
| pmrA-Cheq*^b^* | aacgctggcgaagggtcatcgctc | Δ*pmrA* strain |
| arnA-FRT-Fw | ttttgcctatcacgatatgggatgtcagggggtgcagggtaggctggagctgcttcg | Δ*arnA* strain |
| arnA-FRT-Rv | cgcgatatcgacgctacgcagaaagaagtccagcgtttgggaattagccatggtccatatg | Δ*arnA* strain |
| arnA-Cheq*^b^* | ggtttagcctgtcggtattgcttat | Δ*arnA* strain |
| pmrC-FRT-Fw | gacgcatcaacatgttaaagcgctttcttaaaagacctgtaggctggagctgcttcg | Δ*pmrC* strain |
| pmrC-FRT-Rv | cggttgcagaatatcatctgcggcctggtaatacgtcgtgggaattagccatggtccatatg | Δ*pmrC* strain |
| arn-Fw*^b^* | ccaagcttggaacgatcgcaccgctcgg | pPROBE(NT)::prom*arn* |
| arn-Rv*^b^* | cgggatcccgtgaaagccgcttttcaaatatttcggca | pPROBE(NT)::prom*arn* |
| pmrC-Fw*^b^* | cgggatcccgcgattcggtgacgctaatcgtgaca | pPROBE(NT)::prom*pmrCAB* |
| pmrC-Rv*^b^* | ccaagcttggggctgggttgcttatcgtcggcgt | pPROBE(NT)::prom*pmrCAB* |

*^a^* Restriction sites are underlined.

*^b^* Primer used to confirm the mutant by PCR and sequencing.

**Table S3. SMILES and Labbook codes for compounds used in this study**

| **Labbook Code** | **SMILES** | **Reference** |
| --- | --- | --- |
| CA439 | OC1=CC2=C(NC3=CC=C(OCC4=CC=CC=C4)C=C3)N=CN=C2C=C1 | ^9^ |
| CA176 | C#CC1=CC=CC(NC2=NC=NC3=CC(OC)=C(OC)C=C32)=C1 | ^9^ |
| CA209 | COC1=CC2=NC=NC(NC3=CC=CC(C#C)=C3)=C2C=C1 | ^9^ |
| CA454 | OC1=CC=C2C(NC3=CC=CC(C#C)=C3)=CC=NC2=C1 | ^9^ |
| CA490 | COC1=CC=C(NC2=C3C=C(OC)C=CC3=NC=N2)C=C1 | ^9^ |

Table S4. Checkerboard assays data. Data of the individual assays is provided as a Supplementary Excel document.

**References**

1. Fields, P. I., Swanson, R. V, Haidaris, C. G. & Heffron, F. Mutants of Salmonella typhimurium that cannot survive within the macrophage are avirulent. *Proc Natl Acad Sci U S A* **83**, 5189–93 (1986).

2. Viarengo, G. *et al.* Unsaturated long chain free fatty acids are input signals of the Salmonella enterica PhoP/PhoQ regulatory system. *J Biol Chem* **288**, 22346–58 (2013).

3. Aguirre, A. *et al.* PhoP-induced genes within Salmonella pathogenicity island 1. *J Bacteriol* **188**, 6889–98 (2006).

4. Cardenal-Muñoz, E. & Ramos-Morales, F. DsbA and MgrB regulate steA expression through the two-component system PhoQ/PhoP in Salmonella enterica. *J Bacteriol* **195**, 2368–78 (2013).

5. Seribelli, A. A. *et al.* Phenotypic and genotypic characterization of Salmonella Typhimurium isolates from humans and foods in Brazil. *PLoS One* **15**, e0237886 (2020).

6. Milanez, G. P. *et al.* Whole-Genome Sequence of Salmonella enterica Serovar Enteritidis Phage Type 4, Isolated from a Brazilian Poultry Farm. *Genome Announc* **4**, (2016).

7. Miller, W. G., Leveau, J. H. & Lindow, S. E. Improved gfp and inaZ broad-host-range promoter-probe vectors. *Mol Plant Microbe Interact* **13**, 1243–50 (2000).

8. Datsenko, K. A. & Wanner, B. L. One-step inactivation of chromosomal genes in Escherichia coli K-12 using PCR products. *Proc Natl Acad Sci U S A* **97**, 6640–5 (2000).

9. Carabajal, M. A. *et al.* Quinazoline-Based Antivirulence Compounds Selectively Target Salmonella PhoP/PhoQ Signal Transduction System. *Antimicrob Agents Chemother* **64**, (2019).
